# Larger frogs are better mimics but are more risk-averse in a nontoxic poison frog

**DOI:** 10.1093/beheco/araf117

**Published:** 2025-10-06

**Authors:** Brendan L McEwen, Justin Yeager, Ana Veneat, James B Barnett

**Affiliations:** Psychology, Neuroscience, and Behaviour, McMaster University, Hamilton, ON, Canada L8S 4K1; Grupo de Investigación en Biodiversidad, Medio Ambiente y Salud (BIOMASS), Facultad de Ingenierías y Ciencas Aplicadas, Universidad de las Américas, Quito 170124, Ecuador; Psychology, Neuroscience, and Behaviour, McMaster University, Hamilton, ON, Canada L8S 4K1; School of Natural Sciences, Trinity College Dublin, Dublin 2, D02 PN40, Ireland

**Keywords:** Batesian mimicry, boldness, exploratory behavior, light environment, ontogeny, poison frogs

## Abstract

Aposematic species signal to potential predators with salient and recognizable coloration. Predators learn to associate these warning signals with secondary defenses (eg toxins) and will subsequently avoid attacking aposematic prey. Warning signals can therefore reduce the need to hide and/or flee and alleviate some of the energetic/opportunity costs of predator avoidance. Consequently, aposematic species are frequently active and bold in behavior. Batesian mimics replicate the colors, and often the behavior, of aposematic species and may benefit from a similar reduction in predation risk and energetic/opportunity costs. *Allobates zaparo* (Aromobatidae) is a nontoxic Batesian mimic of the chemically defended poison frog *Ameerega bilinguis* (Dendrobatidae). However, the efficacy of mimicry appears to change throughout ontogeny as *Al. zaparo* develops from a seemingly cryptic juvenile to the mimetic adult. We examined how morphological mimicry (ie color) and the propensity to explore a novel environment (ie boldness) changed throughout ontogeny. We predicted that mimicry would improve with increasing size and that better mimics would engage in more exploratory behavior. We found that larger mimics more closely matched their model however they were less likely to be active than were smaller frogs. These data suggest that larger size, and more accurate mimicry, do not necessarily correspond to increases in behavioral boldness. This result may arise from limitations in Batesian mimicry but factors including foraging requirements or social/reproductive behavior cannot be discounted. More research is needed to understand the relationship between behavior, color, body size, and maturity in these frogs and across Batesian mimics more widely.

## Teaser

Batesian mimics are undefended species which replicate the colors and behaviors of defended models. This allows mimics to avoid the costs of having to hide/flee from predators. *Allobates zaparo* is a Batesian mimic, but the efficacy of mimicry appears to change throughout life. We tested whether the development of mimicry is associated with a corresponding increase in exploratory behavior. We found that mimicry improves throughout growth; however, adults were more risk averse than were juveniles.

## Introduction

Aposematism, in which a defended species evolves a salient signal (eg coloration) to advertise its secondary defenses (eg chemical toxins), produces some of the most striking visual phenotypes in the natural world (Stevens and Ruxton 2012). Predators familiar with these warning signals will then avoid consuming prey displaying such signals in future (Skelhorn and Rowe 2006; Skelhorn et al. 2016; Ruxton et al. 2018). This, in turn, can reduce the risk of predation during conspicuous behaviors which may draw the attention of potential predators (Speed et al. 2010; Ruxton et al. 2018). For example, aposematic signals may allow defended prey to forage more efficiently or to engage in salient social or reproductive behaviors without needing to hide or escape from potential predators. Aposematic signals are therefore often associated with bold behavior, with defended species being more active, less likely to flee from predators, and more likely to explore novel environments than nondefended, or cryptically colored, species (Poulton 1890; Whitman et al. 1985; Hatle and Grimké Faragher 1998; Mappes et al. 2005).

The benefits of aposematism can be parasitized through Batesian mimicry, in which a nondefended “mimic” species co-opts the signal of a sympatric, defended, “model” species (Bates 1862; Pasteur 1982). Predators incorrectly classify the palatable mimic as an unpalatable model and will avoid consuming either prey (Bates 1862; Endler 1981; Ruxton et al. 2018). Moreover, in addition to mimicking morphological traits, mimics may also replicate the behaviors of defended models (Srygley 1999; Page et al. 2024; Tan et al. 2024). This convergence in behavior may arise for 2 primary reasons: (1) mimicking the bold behavior of a defended model can enhance the aversive properties of the visual signal and further diminish predation risk (Paluh et al. 2014), and/or (2) when released from the need to hide or flee from predators, mimics can more conspicuously engage in other behaviors such as foraging, social signaling, or reproduction (Speed et al. 2010).

Indeed, a reduction in the opportunity or energetic costs that arise from extended vigilance, avoidance, or escape behaviors has been suggested to be a driver for the initial evolution of both aposematism and mimicry (Speed and Ruxton 2005; Speed et al. 2010; Ruxton et al. 2018). However, Batesian mimics are frequently considered “imperfect” as they rarely fully replicate all aspects of their model's visual signal (Sherratt 2002; Gilbert 2005; Kikuchi and Pfennig 2013; McLean et al. 2019). As morphological mimicry has been suggested to facilitate the evolution of bold behavior (Page et al. 2024; Tan et al. 2024), this raises the question of how the behavior of mimics will vary depending on visual similarity to their model. For example, we may expect bold and risk-taking behavior to be correlated with morphological mimicry as deviation from perfect mimicry is predicted to increase predation risk and favor the retention of more cautious predator avoidance behavior (Ruxton et al. 2018).

The Neotropical poison frogs (Dendrobatoidea) provide many examples of how color can affect the evolution of behavior and vice versa (Rudh et al. 2013; Rojas 2017; Dugas et al. 2020; Klank et al. 2024; Rojas and Vargas-Salinas 2024; Vargas-Salinas and Rojas 2024; Barnett et al. 2025). Bright warning colors have evolved in tandem with the sequestration of potent alkaloid toxins (Summers and Clough 2001; Santos et al. 2003; Saporito et al. 2007; Maan and Cummings 2012), salient visual and acoustic signals used to attract mates and defend territories (Summers 2000; Maan and Cummings 2008; Crothers and Cummings 2015), and complex parental care, including egg guarding, tadpole transport, and food provision (Ringler et al. 2013; Schulte 2014; Stynoski et al. 2014; Carvajal-Castro et al. 2021). Poison frogs are also frequently active and bold in behavior, having a reduced propensity to flee long distances from approaching predators (Cooper et al. 2009; Pröhl and Ostrowski 2011; Gray et al. 2023; Barnett et al. 2025); behavior which can increase the efficacy of their warning signals (Paluh et al. 2014) and allow frogs to engage in otherwise risky feeding and reproductive activity (Killius and Dugas 2014; Stynoski et al. 2014; Dugas et al. 2015). For example, in the poison frog *Oophaga pumilio* (Dendrobatidae), behavior varies between color morphs, with more brightly colored and toxic populations being more active, aggressive, and more likely to engage in exploratory behaviors (Pröhl and Ostrowski 2011; Rudh et al. 2011, 2013; Dugas et al. 2020).

**Fig. 1.**
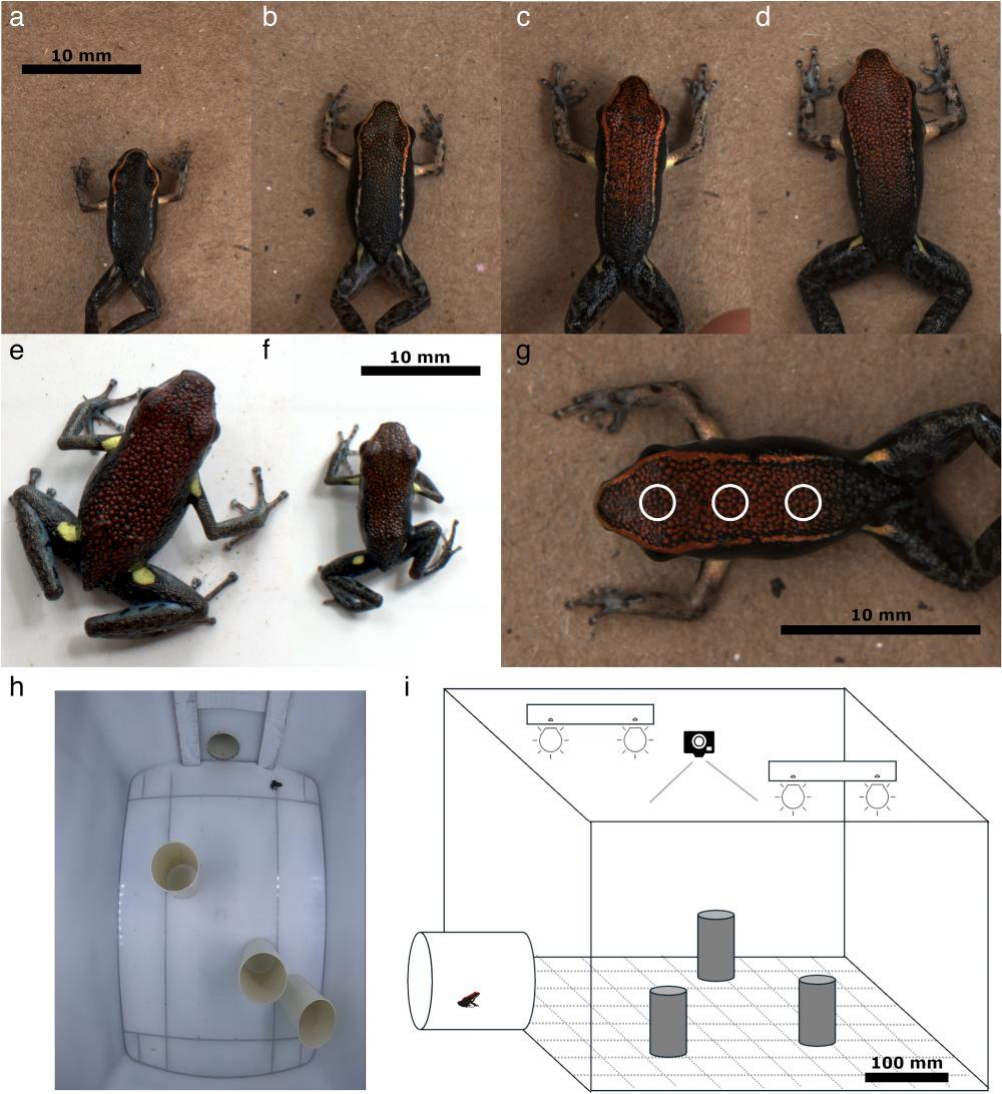
The experimental system. a to d) *Allobates zaparo* undergoes ontogenetic change in color and body size from a seemingly cryptic juvenile a) to a more brightly colored, mimetic adult d). e and f) *Ameerega bilinguis* does not exhibit the same degree of ontogenetic color change as *Al. zaparo* with adults e) and juveniles f) being similar in appearance. g) The placement of the 3 ROIs (D1-3 from anterior to posterior) used to quantify dorsal color, example shows *Al. zaparo*. h and i) The experimental arena, a screenshot from a behavioral trail h) and a schematic i) showing the placement of the acclimatization area, visual barriers, lighting, and camera.

However, not all poison frogs are chemically defended (Summers and Clough 2001; Santos et al. 2003), and *Allobates zaparo* (Aromobatidae) is a nontoxic Batesian mimic of the chemically defended poison frogs *Ameerega bilinguis* and *Ameerega parvula* (Dendrobatidae) (Darst et al. 2006; Darst and Cummings 2006; McEwen et al. 2024). All 3 species share a red dorsum, and domestic chickens (a proxy for wild avian predators) familiar with either model will also avoid *Al. zaparo* (Darst et al. 2006; Darst and Cummings 2006). Moreover, *Al. zaparo* appears to largely mimic the bold behavior of *Am. bilinguis* during close-range encounters with predators (Barnett et al. 2025). However, despite evidence for both morphological and behavioral mimicry in adult *Al. zaparo*, juvenile *Al. zaparo* lack the red coloring indicative of *Am. bilinguis* and *Am. parvula* (Fig. 1a to f). Indeed, upon metamorphosis, *Al. zaparo* has a black dorsum with a tan-yellow dorsolateral ring which is reminiscent of many other nontoxic poison frog species (Amézquita et al. 2017). Yet, throughout development, this dorsolateral ring first shifts toward a copper-orange color and then to red, with red coloring finally spreading from the snout to cover the whole of the dorsum (Fig. 1a to d). In contrast, *Am. bilinguis* and *Am. parvula* do not appear to undergo a similar degree of ontogenetic change (Poelman et al. 2010; McEwen personal observation; Fig. 1e and f).

Ontogenetic changes from cryptic to conspicuous coloring are often associated with changes in the relative efficacy of camouflage and aversive signaling that result from larger body size, or with the increasing need for intraspecific signaling in reproductively active adults (Booth 1990; Higginson and Ruxton 2010; Postema et al. 2023). Correspondingly, adult *Al. zaparo* are significantly larger than juveniles and adult male *Allobates* spp. will conspicuously defend territories where they court potential mates (Ursprung et al. 2011; Chaloupka et al. 2022; Rodríguez et al. 2022). We therefore hypothesized that, in *Al. zaparo*, larger body size and more effective mimicry would be associated with an increase in activity and exploratory behavior. In this study, we first tested this hypothesis with visual modeling in which we compared the colors of *Al. zaparo* to *Am. bilinguis*. We then examined the exploratory behavior of *Al. zaparo* within an unfamiliar environment and under 2 light conditions representing different levels of perceived risk. Specifically, we predicted that mimic fidelity would increase with increasing body size, that frogs would be less active under brighter light, and that, when compared with smaller frogs, larger individuals would be more likely to enter, and more active within, a novel environment.

## Materials and methods

### Sampling

Between 6 July and 5 August 2023, we collected a total of 90 *Al. zaparo*, across a range of different sizes (11.65 to 27.03 mm snout–vent length [hereafter SVL]), and 5 adult *Am. bilinguis* (17.95 to 22.08 mm SVL) at the Iyarina Andes & Amazon Field School, Provincia de Napo, Ecuador (Fig. 1a to e). *Ameerega parvula* was not evident at our field site during data collection. We encountered frogs during daylight hours, along surveys of the field station and forest reserve. The field station grounds are a series of low wooden buildings set amongst patches of secondary rainforest. The forest reserve is a majority secondary rainforest situated ∼2.5 km from the field station. We captured each frog using a 50 ml plastic container, to minimize direct contact. Each frog was then placed into a 1 l plastic bag lined with soil and leaf litter from the capture site and transported to a laboratory at the field station. It was not possible to visually identify the sex of juvenile *Al. zaparo* in situ, and so we did not record the sex of our sample of frogs. Experiments were approved by the McMaster Animal Research Ethics Board (AREB#: 18-05-20) and the Ministerio del Ambiente, Ecuador (permit: MAATE-ARSFC-2022-2694; MAATE-ARSFC-2024-0029).

### Housing and husbandry

At the research station, we individually housed each frog in a 15 cm × 15 cm (diameter × height) cylindrical, opaque, plastic container. Each enclosure was lined with damp paper towel and contained a green leaf to provide humidity and microhabitat structure. We fed frogs ad libitum with termites; prey which form a significant portion of their natural diet (Darst et al. 2005). We misted each enclosure once per day with filtered spring water to maintain humidity and provide hydration. Frogs were released to the site of their initial encounter within 4 d of capture.

### Photography

We photographed each frog with a Nikon D7500 DSLR camera equipped with an AF-S NIKKOR 85 mm lens (Nikon Corp., Japan). Frogs were photographed against a matte cardstock background to minimize specular reflectance and edge artifacts. Photographs were taken from an aerial perspective and under diffuse natural daylight, with the camera held 30 cm above the frog. Each photograph contained a ColorChecker Nano color standard (Calibrite LLC, USA) and a 50 mm scale bar to enable color calibration and scaling. We restricted our photographs to the human-visual spectrum (400 to 700 nm) as previous work on these species suggests that ultraviolet (300 to 400 nm) reflectance is minimal and that the inclusion of UV has minimal effect on measures of signal contrast (Yeager and Barnett 2021; McEwen et al. 2024).

### Visual modeling

To assess how closely the dorsal color of *Al. zaparo* mimicked the dorsal color of adult *Am. bilinguis*, we modeled the visual system of a representative visual predator using the MICA Toolbox v.2.3 (Troscianko and Stevens 2015) in ImageJ v.1.53t (Schneider et al. 2012). As birds are often considered to be common visual predators of poison frogs (Siddiqi et al. 2004; Toledo et al. 2007; Maan and Cummings 2012; Dreher et al. 2015), we modeled an avian visual system (Eurasian blue tit, *Cyanistes caeruleus*; *λ*_max_: UVS = 371, SWS = 448, MWS = 503, LWS = 563, Double = 563 nm; Hart et al. 2000) representative of a wide range of different birds (Ödeen and Håstad 2013).

To characterize dorsal color, we selected 3 regions of interest (hereafter “ROIs”) from each frog: D1, D2, and D3, from the anterior (between the eyes), medial (between the front legs), and posterior (midway between the front and rear legs) sections of the dorsum, respectively (Fig. 1g). Each ROI had a diameter of roughly one-third of the width of the frog and its exact shape was selected to avoid regions of specular reflectance. These 3 ROIs were then combined together into 1 discontinuous ROI, using the “Combine ROIs” function, to provide a single measure of the mean dorsal color of each frog. We then used the receptor-noise-limited visual discrimination model to calculate chromatic (hue, Δ*S*) and achromatic (brightness, Δ*L*) contrast between the dorsal colors of the 2 species (Vorobyev and Osorio 1998). As our photographs did not include UV wavelengths, we calculated chromatic contrast from the SWS, MWS, and LWS single cones and achromatic contrast from the response of the Double cone. We set the illuminant to D65 natural daylight and Weber fractions, an approximation of visual receptor noise, to 0.05 (Vorobyev and Osorio 1998; Siddiqi et al. 2004; Maan and Cummings 2012; McEwen et al. 2024).

This approach calculates chromatic and achromatic contrast in a manner equivalent to the “Just Noticeable Difference” (Vorobyev and Osorio 1998; Walton and Stevens 2018). Here, lower values indicate that 2 colors are likely perceived as being more similar to each other, with contrasts below 1 considered indistinguishable even under ideal viewing conditions, contrasts between 1 and 3 considered “closely matched,” and values above 3 indicating an increasing disparity between the 2 colors (Walton and Stevens 2018). In this manner, we calculated Δ*S* and Δ*L* between each *Al. zaparo* (*n* = 90) and each *Am. bilinguis* (*n* = 5). We then computed the mean dorsal contrast value for each *Al. zaparo* as our metric of mimic fidelity.

### Analyzing ontogenetic color change

To examine whether color contrast between *Al. zaparo* and adult *Am. bilinguis* decreased with the increasing body size of *Al. zaparo* (ie whether mimic fidelity increased throughout growth), we constructed 2 generalized additive models (GAMs) using the *mgcv* v.1.9-3 package in R v.4.3.2 (Wood 2011; R Core Team 2020). GAMs are useful for modeling nonlinear associations without making assumptions about the precise shape of the relationship (Wood 2011). We included chromatic (Δ*S*) and achromatic (Δ*L*) contrast as the response variables of 2 separate models. Both models included a thin-plate-spline “smooth term” of body size (SVL) as the fixed effect and were fit using a Gaussian error distribution. We used the function *gam.check* from the *mgcv* package to assess model fit and examine the residuals, and we used the *summary* function from base R v.4.3.2 to compute *F* tests on the smooth terms. GAMs estimate effective degrees of freedom (edf) as a measure of nonlinearity: an edf of 1 indicates a perfectly linear trend and higher values represent an increasingly complex nonlinear relationship. A *P*-value below 0.05 indicates a significant difference in fit between the model containing the smooth term and a null model.

### Novel environment arena

To examine frog behavior, we created a *Novel Environment Test arena* (Fig. 1h and i). This arena was adapted from a design previously used to quantify activity and boldness in *Allobates femoralis* (Aromobatidae) (Peignier et al. 2022; Bégué et al. 2023); a species closely related to *Al. zaparo*, and in some regions found in sympatry, but not part of the mimicry complex (Grant et al. 2017). The arena consisted of a 50 cm × 30 cm × 40 cm (length × width × height), rectangular, opaque, white plastic box (Sterilite, USA) with a 5 cm × 5 cm square grid marked on the interior floor. One end of the arena was outfitted with a 15 cm × 5 cm (length × diameter), opaque, white PVC pipe at floor level. This acted as an acclimation chamber for the frogs and was separated from the arena interior by a retractable barrier. The arena also included 3 upright PVC cylinders (15 cm × 5 cm, height × diameter) to act as visual obstacles (Peignier et al. 2022). The location of the 3 cylinders was randomly selected at the beginning of each day. Two adjustable LED light bars (Fulen CL01-B, China) were fixed to the inside of the lid to illuminate the arena and provide variable light intensity during the assays. We recorded each assay with a GoPro Hero 9 Black video camera fixed to the center of the arena lid (GoPro, Inc., San Mateo, CA, USA).

### Novel environment behavior assays

We then performed a series of behavioral assays to examine how morphological (ie body size and coloration) and environmental (ie lighting and time of day) factors may affect the exploratory behavior of *Al. zaparo* (*n* = 90). Seven frogs missed 1 trial, and 1 frog missed 2 trials. Each behavioral assay followed a standardized procedure adapted from Peignier et al. (2022) and Begue et al. (2023). The frog was placed into the acclimation chamber and allowed to settle undisturbed for 5 min. After acclimation, the camera was activated and the barrier was removed, allowing the frog access to the arena. The experimenter (BLM) left the room and the frog's behavior was recorded for 15 min.

Each frog was tested twice per day (*round*; 1 and 2 per each *day*) for 2 consecutive days (*day*; 1 and 2), once in each of the mornings (09:00 am to 12:00 pm) and once in each of the afternoons (14:00 pm to 17:00 pm). To examine the role of light intensity, each frog underwent behavioral trials in a *low* light condition (∼60 lux) and a *high* light condition (∼1,150 lux). The first trial of each frog was assigned randomly (ie *low* or *high* light) and then counterbalanced (ie *low* then *high* on day 1, followed by *high* then *low* on day 2, or vice versa). Each behavioral trial involved a single frog, but trials were performed in *batches* (*n* = 14) according to when frogs were captured (6 to 8 frogs per *batch*). Each *batch* completed both days of behavior testing, was photographed, and was then released, before a new *batch* was collected.

Light intensity data collected from around the Iyarina forest reserve confirmed that the 2 light treatments were representative of the frogs’ natural light environment. The *low* light treatment corresponded to light levels found at the morning onset (∼06:30 am) and evening conclusion (∼18:30 pm) of frog activity whereas the *high light* treatment corresponded to light levels found in forested areas during the mid-morning (09:30 am to 12:30 pm) and mid-afternoon (14:00 pm—16:30 pm). See the Supplementary material for further details.

### Behavioral scoring of videos

We analyzed the video footage using BORIS v.8.21.10, an open-source event-logging program (Friard and Gamba 2016). The observer (A.V.) was blind to trial details such as Frog ID, day, round, batch, and light treatment.

We recorded 4 aspects of frog behavior to quantify both *boldness*, defined as the propensity to engage in potentially risky behavior (Réale et al. 2010; Herde and Eccard 2013; Cabrera et al. 2021), and activity, defined as the total distance traveled within an area (Herde and Eccard 2013; Peignier et al. 2022; Bégué et al. 2023).

To quantify *boldness*, we recorded both whether the frog entered the arena (*Entry Probability*, binary data) and, if the frog did enter the arena, the latency in seconds before it did so (*Entry Latency*, continuous data). To quantify *activity*, for those individuals which did enter the arena, we recorded the total number of jumps and the number of 5 cm × 5 cm squares that the frog crossed on the arena floor. A frog was considered to have crossed a square when its whole body had passed the boundary from 1 grid square to another. We focused our analysis on terrestrial exploratory behavior and did not include instances when the frogs climbed up the enclosure walls. To account for instances when frogs jumped onto the walls of the arena, we calculated the number of jumps and squares crossed per minute of terrestrial activity (*Jumps/min* and *Squares/min*, continuous data).

### Analyzing behavior

**Fig. 2.**
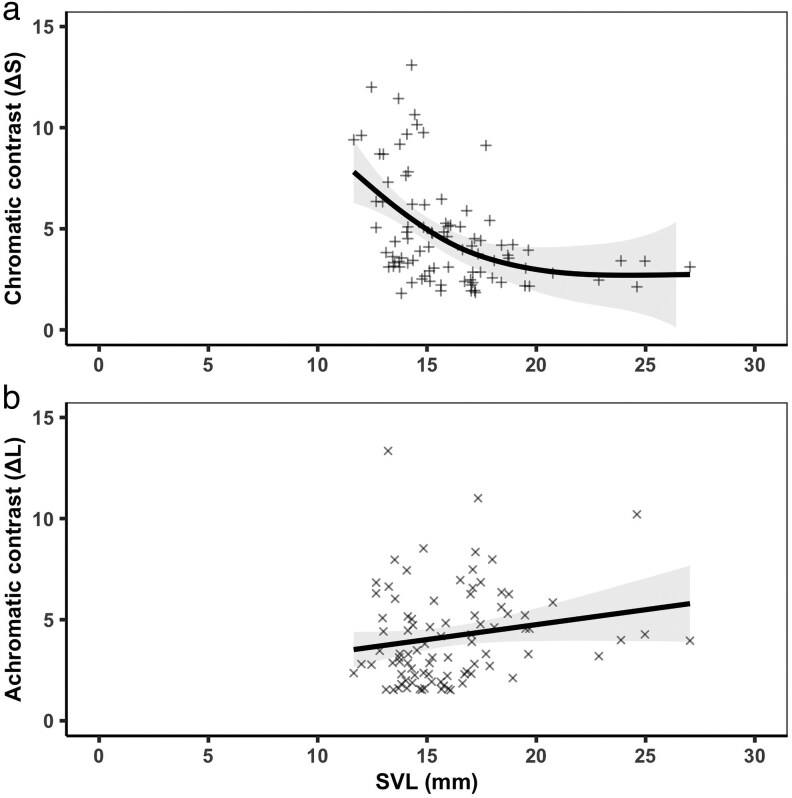
Visual contrast (GAM smoothing curves ± 95% CI from the model) between the dorsal colors of variously sized *Allobates zaparo* (SVL = *Al. zaparo* snout–vent length in mm) and adult *Ameerega bilinguis*. Lower contrast values are indicative of closer mimicry. a) Chromatic contrast (Δ*S*) significantly decreases as *Al. zaparo* increases in size (SVL). b) Achromatic contrast (Δ*L*) does not significantly change throughout the growth of *Al. zaparo* (SVL).

We constructed a series of generalized linear mixed effects models to analyze how body size, color development, and lighting conditions affected frog behavior: ie *Entry Probability*, *Entry Latency*, *Jumps/min*, and *Squares/min*. Frogs which did not enter the arena were excluded from our analyses of *Entry Latency*, *Jumps/min*, and *Squares/min.* Visual inspection of the frog coloration data suggested that there was considerable variance in coloration within a given range of body size and vice versa (Fig. 2). We therefore included both body size (SVL; mm) and color contrast (Δ*S* and Δ*L*) to each model to distinguish between the any effects coloration and body size may have independently had on behavior. We also included the fixed effect of time of day to account for any temporal differences between our experimental sessions. Model inspection through the *check_collinearity* function from the R package *performance* v.0.14.0 (Lüdecke et al. 2021) revealed no adverse effects of collinearity between body size and coloration.

Each model included the fixed effects of chromatic contrast (Δ*S*), achromatic contrast (Δ*L*), body size (SVL in mm), time of day (*morning* or *afternoon*), and light intensity (*high* or *low*). We also included the random effects of Frog ID and the experimental session (ie *round* nested within *day*, nested within *batch*). All models were constructed in R v.4.3.2 (R Core Team 2020) using R package *glmmTMB* v.1.1.11 (Brooks et al. 2017). We checked model assumptions using R packages *DHARMa* v.0.4.7 (Hartig 2025) and *performance* v.0.14.0 (Lüdecke et al. 2021), and to calculate the test statistics for each fixed effect within the model we used the *Anova* function from R package *car* v.3.1-3 (Fox and Weisberg 2019). We log-transformed the *Jumps/min* and *Squares/min* data, and adjusted the error distribution of each model, to improve model fit and adhere to assumptions regarding the distribution of residuals: *Entry Probability* = binomial, *Entry Latency* = Gamma with log-link, log-transformed *Jump/min* = Gaussian, and log-transformed *Squares/min* = Gaussian. If more than 1 main effect was significant (*P* < 0.05), we created an additional model that included an interaction between these factors. We examined the significance of this interaction by comparing between the additive and interactive models using the *anova* function from base R v.4.3.2.

## Results

### Ontogenetic color change

When analyzing the relationship between body size and visual mimicry, we found that as *Al. zaparo* increased in size its dorsal color became a closer match to the dorsal color of adult *Am. bilinguis* (Fig. 2). Specifically, we found that as the body size (SVL) of *Al. zaparo* increased, the chromatic contrast (Δ*S*) between the dorsal colors of *Al. zaparo* and adult *Am. bilinguis* decreased in a nonlinear fashion (Δ*S*: *F* = 9.25, edf = 2.42, *P* < 0.001; Fig. 2a). However, we found no such relationship for achromatic contrast (Δ*L*: *F* = 3.06, edf = 1.00, *P* = 0.084; Fig. 2b).

### Behavioral study

#### Entry probability

**Fig. 3.**
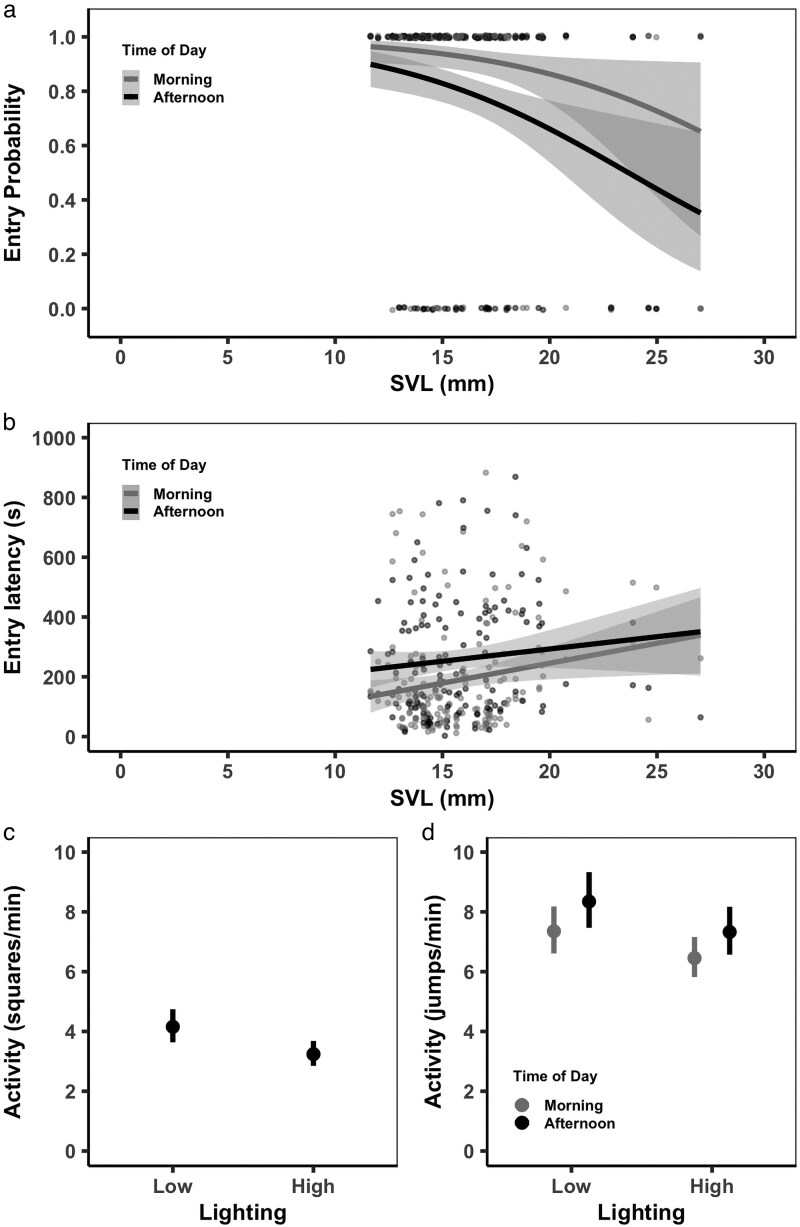
Behavioral responses of variously sized *Allobates zaparo* (SVL = *Al. zaparo* snout-vent length in mm) within the *Novel Environment Test* arena. Frogs which did not enter the arena were excluded from the analysis of *Entry Latency* b) and *Activity* c and d). a) The probability of entering the arena (*Entry Probability*, mean estimated probability ± 95% CI from the model) significantly decreased as the size of *Al. zaparo* (SVL) increased and all frogs were more likely to enter the arena in the afternoon (dark gray) than in the morning (light gray). b) The time taken to enter the arena (*Entry Latency,* mean estimated latency ± 95% CI from the model) significantly increased as the size of *Al. zaparo* (SVL) increased, and all frogs took longer to enter the arena in the afternoon (dark gray) than in the morning (light gray). For *Entry Probability* a) and *Entry Latency* b), there was no significant effect of frog color or light intensity. c) *Activity*, the number of squares crossed within the arena (*Squares/min*, means ± 95% CI from the model) was significantly higher under *low* than under *high* light conditions, but there was no significant effect of frog color, body size, or time of day on the number of squares crossed. d) *Activity*, the number of jumps (*Jumps/min*, means ± 95% CI from the model) was significantly higher in *low* than in *high* lighting conditions, and higher in the afternoon (dark gray) than in the morning (light gray). There was no significant effect of frog color or body size on the number of jumps.

Frogs entered the arena during ∼85% of the behavioral trials (299/351). We found no significant relationship between *Entry Probability* and frog coloration (chromatic contrast [Δ*S*]: *χ*^2^ < 0.01, df = 1, *P* = 0.959; achromatic contrast [Δ*L*]: *χ*^2^ = 0.51, df = 1, *P* = 0.474) nor between *Entry Probability* and light intensity (*χ*^2^ = 2.96, df = 1, *P* = 0.086). However, we did find a significant relationship between *Entry Probability* and body size, with larger frogs being less likely to enter the arena than smaller frogs (*χ*^2^ = 8.37, df = 1, *P* = 0.004; Fig. 3a). We found a significant effect of time of day such that frogs were less likely to enter during the afternoon than in the morning (*χ*^2^ = 11.30, df = 1, *P* < 0.001; Fig. 3a). We also found that the inclusion of the interaction between body size and time of day did not improve model fit (*χ*^2^ = 0.04, df = 1, *P* = 0.836). These results indicate that larger frogs were less likely to enter the arena than smaller frogs, and that all frogs, regardless of body size, were less likely to enter the arena during the afternoon.

#### Entry latency

We found that the relationships between *Entry Latency* and both coloration and light intensity were nonsignificant (chromatic contrast [Δ*S*]: *χ*^2^ = 0.69, df = 1, *P* = 0.406; achromatic contrast [Δ*L*]: *χ*^2^ = 0.56, df = 1, *P* = 0.456; light intensity: *χ*^2^ = 0.72, df = 1, *P* = 0.395). However, we did find that larger frogs took longer to enter the arena than smaller frogs (*χ*^2^ = 4.43, df = 1, *P* = 0.035; Fig. 3b) and that all frogs took longer to enter the arena in the afternoon compared with the morning (*χ*^2^ = 13.21, df = 1, *P* < 0.001; Fig. 3b). Including the interaction between body size and time of day did not improve model fit (*χ*^2^ = 1.68, df = 1, *P* = 0.194). Together these results indicate that, of the frogs that did enter the arena, larger frogs took longer to enter than smaller frogs, and all frogs that entered took longer to do so during the afternoon compared with the morning.

#### Activity—squares crossed

When analyzing the number of squares entered by each frog we focused on the behavior of “active” individuals by excluding trials where a frog did not enter the arena (*n* = 52). We found there to be no significant relationship between *Squares/min* and color contrast (chromatic contrast [Δ*S*]: *χ*^2^ = 0.87, df = 1, *P* = 0.350; achromatic contrast [Δ*L*]: *χ*^2^ = 0.01, df = 1, *P* = 0.916), nor between *Squares/min* and body size (*χ*^2^ = 0.40, df = 1, *P* = 0.526). There was also no difference in the number of squares entered between the morning and afternoon testing periods (*χ*^2^ = 1.62, df = 1, *P* = 0.203). However, we did find that frogs were less active in the *high* light condition than in the *low* light condition (*χ*^2^ = 14.26, df = 1, *P* < 0.001; Fig. 3c). Taken together, these data suggest that the frogs which entered the arena moved through fewer squares during *high* light conditions than *low* light conditions, regardless of color, body size, or the time of day.

#### Activity—jumps performed

For the number of jumps performed by frogs on the arena floor (excluding the 52 trials where the frog did not enter the arena), there was no significant relationship between *Jumps/min* and color contrast (chromatic contrast [Δ*S*]: *χ*^2^ = 2.54, df = 1, *P* = 0.111; achromatic contrast [Δ*L*]: *χ*^2^ = 0.56, df = 1, *P* = 0.455), and no significant effect of body size (*χ*^2^ = 2.73, df = 1, *P* = 0.099). However, frogs performed fewer jumps in the *high* light condition than in the *low* light condition (*χ*^2^ = 5.32, df = 1, *P* = 0.021; Fig. 3d) and fewer jumps in the morning than in the afternoon (*χ*^2^ = 5.07, df = 1, *P* = 0.024; Fig. 3d). Including an interaction between time of day and light treatment did not improve model fit (*χ*^2^ = 0.261, df = 1, *P* = 0.609). Together these results suggest that the frogs which entered the arena jumped more frequently both in the afternoon and when light levels were lower, regardless of their size or coloration (Fig. 3d).

## Discussion

Here, we examined whether ontogenetic changes in morphology (ie coloration and body size) and changes to the light environment (ie light intensity) were associated with changes in the risk-taking behavior and activity levels of the Batesian mimic *Al. zaparo*. We predicted that mimic fidelity would increase throughout growth and, in parallel with the development of the mimetic signal, that larger frogs would be more active and more likely to explore a novel environment.

We indeed found that mimic fidelity did increase with increasing body size, with the dorsal colors of larger *Al. zaparo* being a closer match to adult *Am. bilinguis*. However, contrary to our predictions, we found that smaller frogs were more likely to enter the novel arena, and among the frogs which did enter the arena, smaller frogs did so more quickly. For frogs that entered the arena, activity levels did not differ according to body size. We did, however, find that there was a significant effect of time of day and of light intensity that was not associated with either frog color or body size. All *Al. zaparo* were less likely to enter the arena, and took longer to do so, in the afternoon, performed fewer jumps in the morning, and were less active in *high* light than in *low* light conditions. Therefore, as larger frogs were less likely to enter the arena it was smaller frogs which were more active and more likely to engage in risk-taking behaviors in an unfamiliar environment.

Taken together, our data suggest that although visual similarity to the model increased throughout ontogeny, boldness and activity levels decreased as frogs grew larger. We had predicted that as the development of the mimetic signal should reduce the risk of mortality even upon detection by predators, and because adult *Allobates* spp. engage in conspicuous social behaviors (Ringler et al. 2013), larger individuals would be less risk averse relative to smaller individuals lacking mimetic signals. However, our results instead suggest that the interaction between ontogeny and behavior is more complex, and may also be affected by the additional influence of changes in factors such as detectability, nutritional requirements, and/or reproductive and social behavior.

In a recent study, we found that although the dorsal red color of both *Al. zaparo* and *Am. bilinguis* acts as an aversive signal (Darst and Cummings 2006), it can also provide camouflage against the leaf litter found within the frogs’ natural habitat (McEwen et al. 2024). Poison frogs are not immune from predation (Master 1999; Toledo et al. 2007), and this interaction between aposematism and camouflage likely represents a trade-off between the benefits of having salient, aversive, signals and of remaining undetected by potential predators (Stevens 2007; Kikuchi et al. 2023; Postema et al. 2023). Explorative behavior may experience a similar trade-off. Smaller and less saliently colored juveniles may be better able to hide amongst the leaf litter and escape detection (Barnett et al. 2023; Yu et al. 2024). Larger-bodied adults, on the other hand, may compensate for their increased morphological saliency with less conspicuous behavior.

Alternatively, reproductive pressures such as intrasexual conflict and/or courtship in adult frogs may drive ontogenetic changes to coloration and behavior. Adult male *Al. zaparo* defend territories and so may be less likely to explore beyond their own patch compared with growing juveniles who may also be dispersing away from tadpole deposition sites (Chaloupka et al. 2022; Bégué et al. 2023; Peignier et al. 2023). Reduced adult exploratory behaviors may be especially favored if there is a heightened threat of intraspecific aggression upon entering the territory of a rival (Ursprung et al. 2011; Chaloupka et al. 2022; Rodríguez et al. 2022). Conversely, the mimetic colors in *Al. zaparo* could be the result of co-opting visual signals developed for sexual selection and assortative mate choice, which is the case for some other poison frogs (Maan and Cummings 2008; Gade et al. 2016; Yang et al. 2016). It is currently unknown to what extent sexual selection may have influenced color evolution in *Al. zaparo*. If salient coloring initially evolved in response to sexual selection, and only secondarily for mimicry, then only adults would benefit from having bright coloration. Mimicry and more cautious behavior may have then arisen in response to compensate for the greater risks associated with bright signals primarily directed at conspecifics. While this alternative is perhaps less likely compared with mimicry related hypotheses, future work is needed to investigate the role of color in social communication and how behavior may differ between reproductively mature males and females.

It is also important to note that although we found a significant correlation between body size and coloration, we did not find any direct associations between color and behavior. Behavioral change may therefore predominantly arise due to factors other than visual ecology. For example, juveniles may be selected to enter potentially risky environments if they are under greater resource stress than adults (Arenz and Leger 2000; Brown and Braithwaite 2004; Ward et al. 2004), or if they would benefit from enhanced resource uptake which may affect carotenoid pigment sequestration, increase growth rates, or shorten the time taken to reach reproductive maturity (Flores et al. 2013; Bégué et al. 2024). Similarly, our results may emerge at the population level due to individual differences in pace-of-life syndromes and a foraging-mortality trade-off (Mangel and Stamps 2001; Stamps 2007). In such a scenario, more active individuals may benefit from increased growth rates or earlier reproductive success but experience increased mortality at smaller sizes (Mangel and Stamps 2001; Stamps 2007). Mimicry remains a viable, albeit seemingly indirect, factor underlying changes to behavior, but more research is required to tease apart the relationship between color, body size, and maturity, and how the rate of change may be differentially influenced by factors such as resource acquisition.

Taken together our data suggest that the ontogenetic development of *Al. zaparo* is indeed characterized by a significant shift in coloration, from seemingly cryptic to saliently mimetic. This increase in morphological salience is however accompanied by a significant reduction in bold exploratory behavior. Previous work suggests that Batesian mimicry can facilitate increasingly bold behavior by reducing the threat of predation even upon detection by a would-be predator (Page et al. 2024; Barnett et al. 2025). However, here we found the opposite effect, with better mimics being more risk-averse. This may suggest that for undefended Batesian mimics, cautious behavior can compensate for the risks of increasing detectability. Alternately, other factors which develop along with color, such as increasing body size and reaching sexual maturity, may have a greater impact on behavior. Future work is therefore necessary to examine how the interacting effects of different defensive and reproductive strategies change throughout the lifecycle of *Al. zaparo*, the potential role of coloration in sexual selection, and how such trade-offs between visual signals and behavior may affect Batesian mimics more widely.

## Supplementary Material

araf117_Supplementary_Data

## Data Availability

Analyses reported in this article can be reproduced using the data provided by McEwen et al. (2025).
