# Supplementary material for: Larger frogs are better mimics but are more risk-averse in a nontoxic poison frog
Source: Behav Ecol. 2025 Oct 6;36(6):araf117. doi: 10.1093/beheco/araf117 (PMC12596420; doi:10.1093/beheco/araf117)
Supplement: araf117_Supplementary_Data [file araf117_supplementary_data.zip › McEwen-etal-2025_Rmarkdown.pdf]

# Analysis 1 - Colour contrast analysis

McEwen BL, Yeager J, Veneat A, & Barnett JB

2025-06-23

## Analysis 1 - Colour contrast analysis

Here we will assess how closely Allobates zaparo (a non-toxic Batestian mimic) mimics the dorsal colours of Ameerega bilinguis (a chemically defended model), And how the efficacy of mimicry changes with the size of Al. zaparo

In this Rmarkdown script we will analyse how chromatic & achromatic contrast between Al. zaparo & adult Am. bilinguis changes according to the size of Al. zaparo.

dS - chromatic contrast (hue) between Al. zaparo & adult Am. bilinguis dL- achromatic contrast (brightness) between Al. zaparo & adult Am. bilinguis SVL - body length of Al. zaparo (snout-vent length)

We have 90 individuals of Al. zaparo (of various ages/sizes) Each Al. zaparo was compared to 5 adult Am. bilinguis Measures of dS and dL reported here are mean values, calculated across the 5 Am. bilinguis for each Al. zaparo

Variables included in this analysis:

FocalFrog = unique alphanumeric code assigned to each individual Al. zaparo

vids = unique alphanumeric code assigned to each individual video file

SVL = the length of the frog in mm

DorsalColJND = chromatic contrast (dS)

DorsalLumJND = achromatic contrast (dL)

```
# First, loading the required packages:
library(tidyverse) # Organizing the data
```

```
## -- Attaching core tidyverse packages ------------------------ tidyverse 2.0.0 --
## v dplyr     1.1.4     v readr     2.1.5
## v forcats   1.0.0     v stringr   1.5.1
## v ggplot2   3.5.2     v tibble    3.3.0
## v lubridate 1.9.4     v tidyr     1.3.1
## v purrr     1.0.4
## -- Conflicts ------------------------------------------ tidyverse_conflicts() --
## x dplyr::filter() masks stats::filter()
## x dplyr::lag()    masks stats::lag()
## i Use the conflicted package (<http://conflicted.r-lib.org/>) to force all conflicts to become errors
```

```
library(mgcv) # Analyzing the data with generalized additive model (GAMs)
```

```
## Loading required package: nlme
##
## Attaching package: 'nlme'
##
## The following object is masked from 'package:dplyr':
```

```
##
##     collapse
##
## This is mgcv 1.9-3. For overview type 'help("mgcv-package")'.
```

```
library(tidygam) # Plotting the GAM prediction
library(tidymv) # Plotting the GAM prediction
```

```
## tidymv has been superseded by tidygam. The package tidymv is no longer maintained but will be
##     kept on CRAN to ensure reproducibility of older analyses. Users should
##     use the replacement package tidygam for new analyses, which is available on
##     CRAN and GitHub (https://github.com/stefanocoretta/tidygam).
##
## Attaching package: 'tidymv'
##
## The following objects are masked from 'package:tidygam':
##
##     get_difference, predict_gam
```

```
library(ggplot2) # Plotting the data
library(patchwork) # Combining plots into a multifaceted figure

# Clear the work space
rm(list=ls())
```

### Read in and check the data

```
# Read in all of the colour contrast & behaviour data
dat <- read.csv("MS_BehaviourData.csv", stringsAsFactors = T)

# We don't need all of the behaviour data
# so we can just select the variables needed for this analysis
dat <- dat %>%
  select(FocalFrog, vids, SVL, DorsalColJND, DorsalLumJND)

# The experiment included repeated measures for the behaviour trials
# consequently there are multiple rows for each frog (one per vid)
# This means that there are several (identical) copies of the
# colour contrast data for each frog
# we need to select just one copy per individual
single.SVL.contrastvals <- dat %>%
  group_by(FocalFrog) %>%
  slice_min(order_by = vids)

# convert the tibble into a data frame
single.SVL.contrastvals <- as.data.frame(single.SVL.contrastvals)

# Check that we have the correct number of data points
nrow(single.SVL.contrastvals)
```

```
## [1] 90
```

```
# There are 90 rows (one per frog ID)
nlevels(single.SVL.contrastvals$FocalFrog)
```

```
## [1] 90
```

```
# And 90 individual frog IDs (FocalFrog)
# This is correct
```

### Plot the raw data

```
# Plot the raw chromatic contrast data
  ggplot(data = single.SVL.contrastvals) +
  geom_point(aes(x = SVL, y = DorsalColJND)) +
  theme_bw() +
  theme(panel.grid.major = element_blank()) +
  theme(panel.grid.minor = element_blank()) +
  ylab ("Chromatic contrast (dS)") +
  xlab ("Frog size (SVL mm)") +
  theme(legend.title = element_blank()) +
  theme(legend.background = element_rect(linetype="solid", colour ="black"))
```

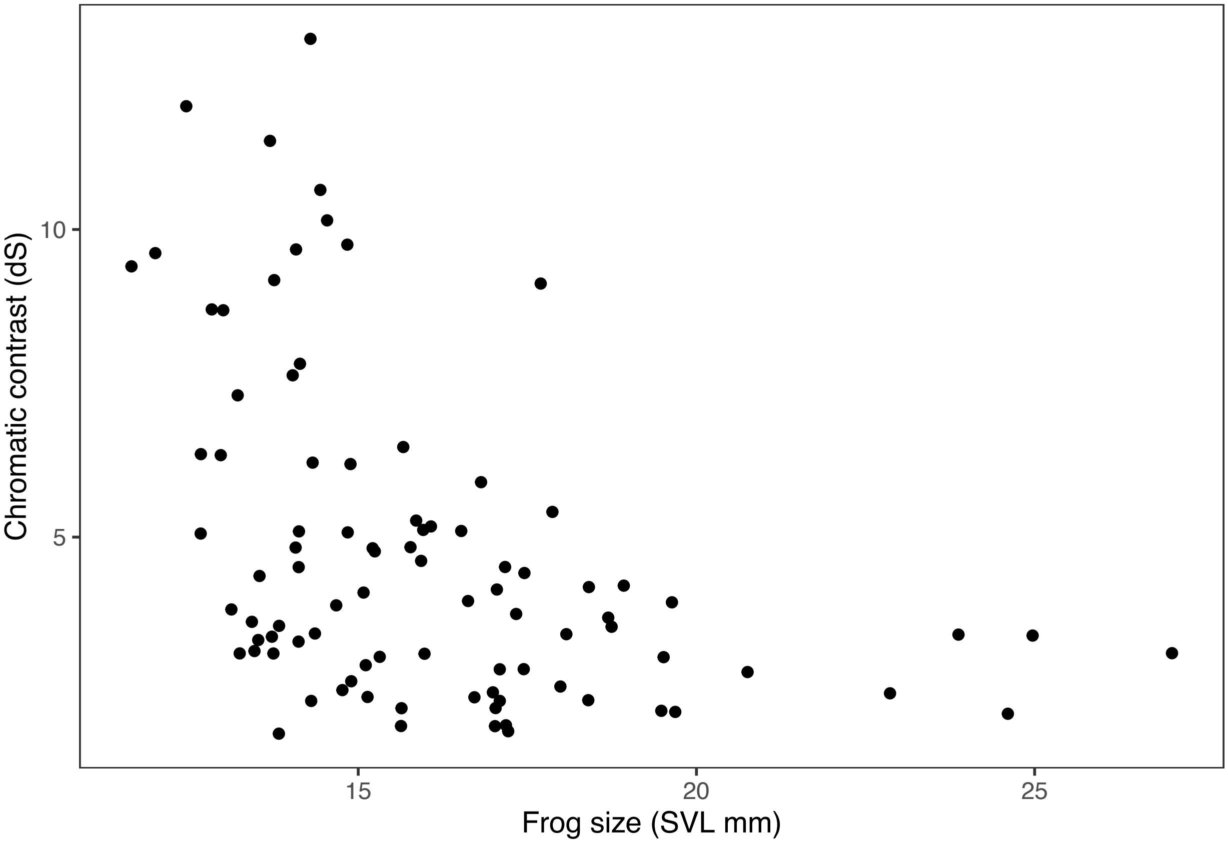


```
# Plot the raw chromatic contrast data
  ggplot(data = single.SVL.contrastvals) +
  geom_point(aes(x = SVL, y = DorsalLumJND)) +
  theme_bw() +
  theme(panel.grid.major = element_blank()) +
  theme(panel.grid.minor = element_blank()) +
  ylab ("Chromatic contrast (dS)") +
  xlab ("Frog size (SVL mm)") +
  theme(legend.title = element_blank()) +
  theme(legend.background = element_rect(linetype="solid", colour ="black"))
```

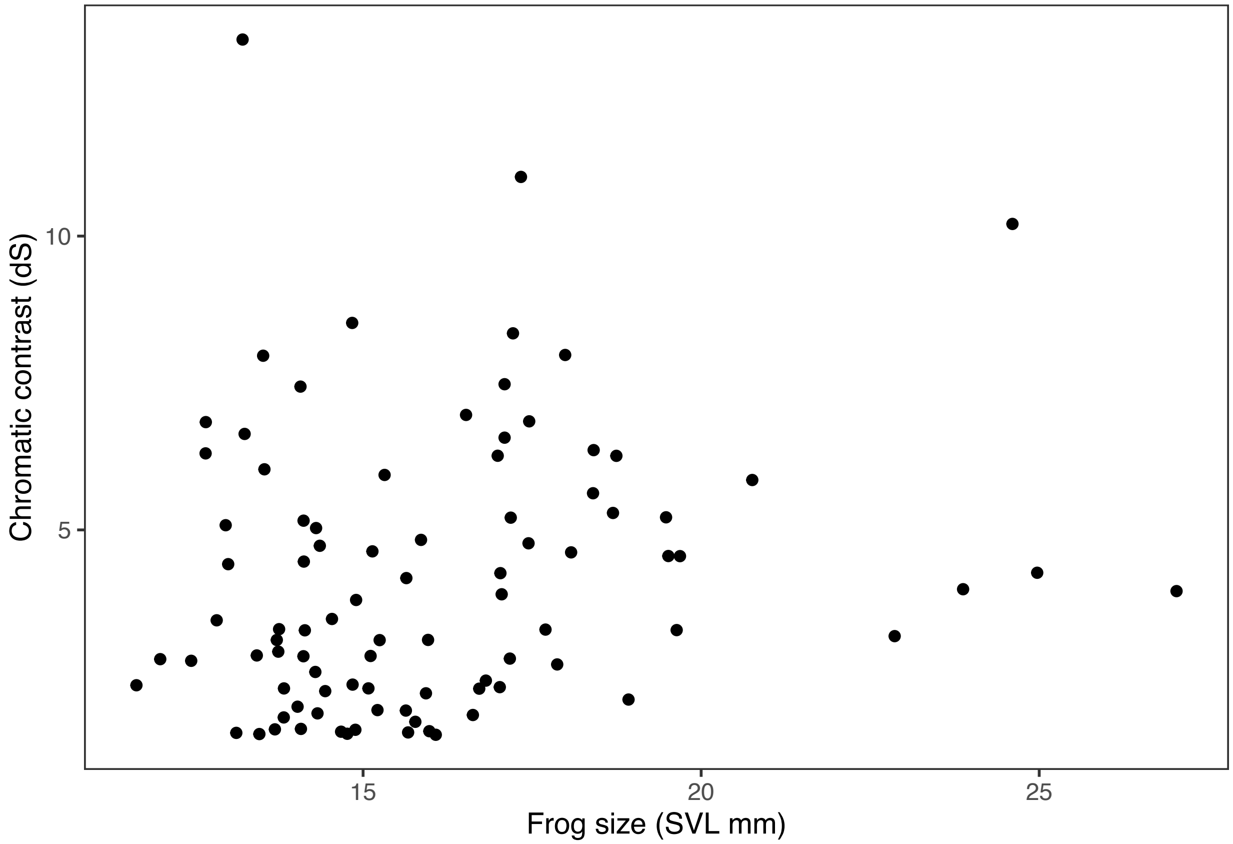


**We can now analyse the data**

**Both chromatic and achromatic contrast may be non-linear**

**So we use GAMs to analyse the relationship between contrast & body size**

```
# The GAM includes chromatic contrast (DorsalColJND) as the response variable
# And the smooth term of body size (SVL)
dorsalcolgam <- gam(DorsalColJND ~ s(SVL),
                    data = single.SVL.contrastvals,
                    family = gaussian,
                    method = "REML")

# check model assumptions using the function gam.check
# chromatic contrast
gam.check(dorsalcolgam)
```

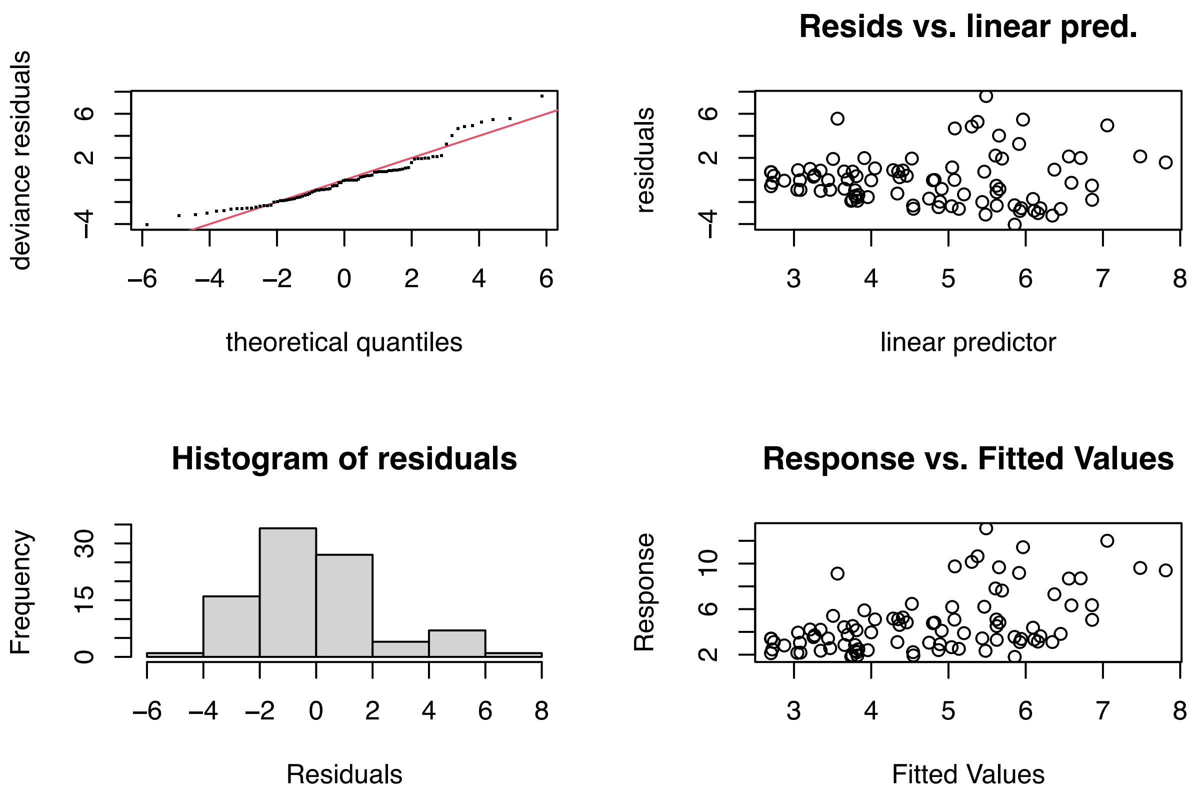


```
##
## Method: REML   Optimizer: outer newton
## full convergence after 6 iterations.
## Gradient range [-2.540887e-09,2.041745e-11]
## (score 204.1784 & scale 5.327166).
## Hessian positive definite, eigenvalue range [0.579141,44.01168].
## Model rank =  10 / 10
##
## Basis dimension (k) checking results. Low p-value (k-index<1) may
## indicate that k is too low, especially if edf is close to k'.
##
##           k'  edf k-index p-value
## s(SVL) 9.00 2.42    1.03    0.55
```

```
# All tests are non-significant (p > 0.05)
# We can reasonably conclude that the model fits the data well,
# and conforms to all assumptions.
```

```
# The GAM includes achromatic contrast (DorsalLumJND) as the response variable
# And the smooth term of body size (SVL)
dorsallumgam <- gam(DorsalLumJND ~ s(SVL),
                    data = single.SVL.contrastvals,
                    family = gaussian,
                    method ="REML")

gam.check(dorsallumgam)
```

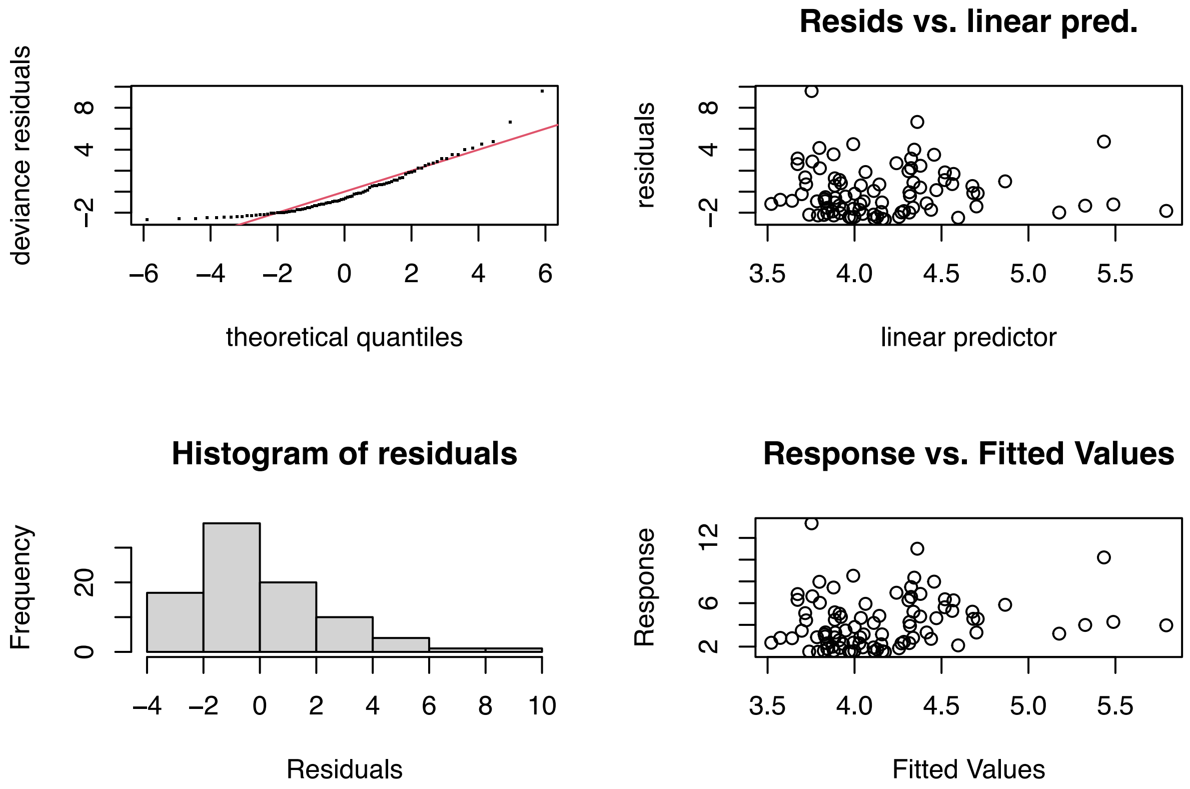


```
##
## Method: REML   Optimizer: outer newton
## full convergence after 9 iterations.
## Gradient range [-4.569883e-05,2.865386e-05]
## (score 203.5216 & scale 5.394312).
## Hessian positive definite, eigenvalue range [4.569155e-05,43.99997].
## Model rank =  10 / 10
##
## Basis dimension (k) checking results. Low p-value (k-index<1) may
## indicate that k is too low, especially if edf is close to k'.
##
##          k' edf k-index p-value
## s(SVL)  9   1    0.99    0.46
```

```
# All tests are non-significant (p > 0.05)
# We can reasonably conclude that the model fits the data well,
# and conforms to all assumptions.
```

```
# Model results for chromatic contrast
summary(dorsalcolgam)
```

```
##
## Family: gaussian
## Link function: identity
##
## Formula:
## DorsalColJND ~ s(SVL)
##
## Parametric coefficients:
##              Estimate Std. Error t value Pr(>|t|)
```

```
## (Intercept)   4.7415     0.2433   19.49   <2e-16 ***
## ---
## Signif. codes:  0 '***' 0.001 '**' 0.01 '*' 0.05 '.' 0.1 ' ' 1
##
## Approximate significance of smooth terms:
##          edf Ref.df     F  p-value
## s(SVL) 2.424  3.033 9.247 2.14e-05 ***
## ---
## Signif. codes:  0 '***' 0.001 '**' 0.01 '*' 0.05 '.' 0.1 ' ' 1
##
## R-sq.(adj) =  0.234   Deviance explained = 25.5%
## -REML = 204.18  Scale est. = 5.3272    n = 90
```

```
# There is a significant relationship between dS & SVL (F = 9.25, p < 0.001)
# This relationship is non-linear (edf = 2.42)
```

```
# Model results for achromatic contrast
summary(dorsallumgam)
```

```
##
## Family: gaussian
## Link function: identity
##
## Formula:
## DorsalLumJND ~ s(SVL)
##
## Parametric coefficients:
##             Estimate Std. Error t value Pr(>|t|)
## (Intercept)   4.1701     0.2448   17.03   <2e-16 ***
## ---
## Signif. codes:  0 '***' 0.001 '**' 0.01 '*' 0.05 '.' 0.1 ' ' 1
##
## Approximate significance of smooth terms:
##        edf Ref.df     F p-value
## s(SVL)   1      1 3.062  0.0836 .
## ---
## Signif. codes:  0 '***' 0.001 '**' 0.01 '*' 0.05 '.' 0.1 ' ' 1
##
## R-sq.(adj) =  0.0227   Deviance explained = 3.36%
## -REML = 203.52  Scale est. = 5.3943    n = 90
```

```
# The relationship between dL & SVL is non-significant (F = 3.06, p = 0.084)
# This trend marginal and the relationship is linear (edf = 1.00)
```

### Plotting the data

```
# Format the data for the plot
# extract GAM prediction for plotting

# Chromatic contrast
GAM_dS <- as.data.frame(predict_gam(dorsalcolgam))

# Achromatic contrast
GAM_dL <- as.data.frame(predict_gam(dorsallumgam))
```

```r
# Plot the data using ggplot2
# including the raw data (from single.SVL.contrastvals)
# and the estimated GAM smooth term from GAM_dS +/- 95% CI (from GAM_dS/GAM_dL)

# Chromatic contrast plot
Colplot <-
  ggplot(data = GAM_dS,
         aes(x = SVL, y = fit)) +
  geom_point(data = single.SVL.contrastvals,
             aes(x = SVL, y = DorsalColJND),
             color = "black",
             alpha = 0.5,
             shape = 3,
             size = 1) +
  geom_smooth_ci() +
  geom_line(color = "black", linewidth = 1) +
  labs(x = "SVL (mm)",
       y = "Chromatic contrast (dS)")+
  scale_x_continuous(limits = c(0, 30), breaks = seq(0, 30, 5)) +
  scale_y_continuous(limits = c(0, 15), breaks = seq(0, 15, 5)) +
  theme_bw() +
  theme(axis.line = element_line(colour = "black"),
        panel.grid.major = element_blank(),
        panel.grid.minor = element_blank(),
        panel.background = element_blank()) +
  theme(panel.grid = element_blank()) +
  theme(panel.grid = element_blank()) +
  theme(plot.title = element_blank()) +

  theme(axis.title.y = element_text(size = 9, face = "bold")) +
  theme(axis.title.x = element_blank()) +
  theme(axis.text.x = element_text(size = 8, face = "bold")) +
  theme(axis.text.y = element_text(size = 8, face = "bold")) +
  theme(axis.ticks.y = element_line(linewidth = 0.7)) +
  theme(axis.ticks.x = element_line(linewidth = 0.7))

# Achromatic contrast plot
Lumplot <-
  ggplot(data = GAM_dL,
         aes(x = SVL, y = fit)) +
  geom_point(data = single.SVL.contrastvals,
             aes(x = SVL, y = DorsalLumJND),
             color = "black",
             alpha = 0.5,
             shape = 4,
             size = 1) +
  geom_smooth_ci() +
  geom_line(color = "black", linewidth=1) +
  labs(x = "SVL (mm)",
       y = "Achromatic contrast (dL)")+
  scale_x_continuous(limits = c(0, 30), breaks = seq(0, 30, 5)) +
  scale_y_continuous(limits = c(0, 15), breaks = seq(0, 15, 5)) +
  theme_bw() +
```

```
  theme(axis.line = element_line(colour = "black"),
        panel.grid.major = element_blank(),
        panel.grid.minor = element_blank(),
        panel.background = element_blank()) +
  theme(panel.grid = element_blank()) +
  theme(panel.grid = element_blank()) +
  theme(plot.title = element_blank()) +

  theme(axis.title.y = element_text(size = 9, face = "bold")) +
  theme(axis.title.x = element_text(size = 9, face = "bold")) +
  theme(axis.text.x = element_text(size = 8, face = "bold")) +
  theme(axis.text.y = element_text(size = 8, face = "bold")) +
  theme(axis.ticks.y = element_line(linewidth = 0.7)) +
  theme(axis.ticks.x = element_line(linewidth = 0.7))

# Combining the plots together into one multifaceted figure
combined_plot <- Colplot + Lumplot +
  plot_layout(nrow = 2)
print(combined_plot)
```

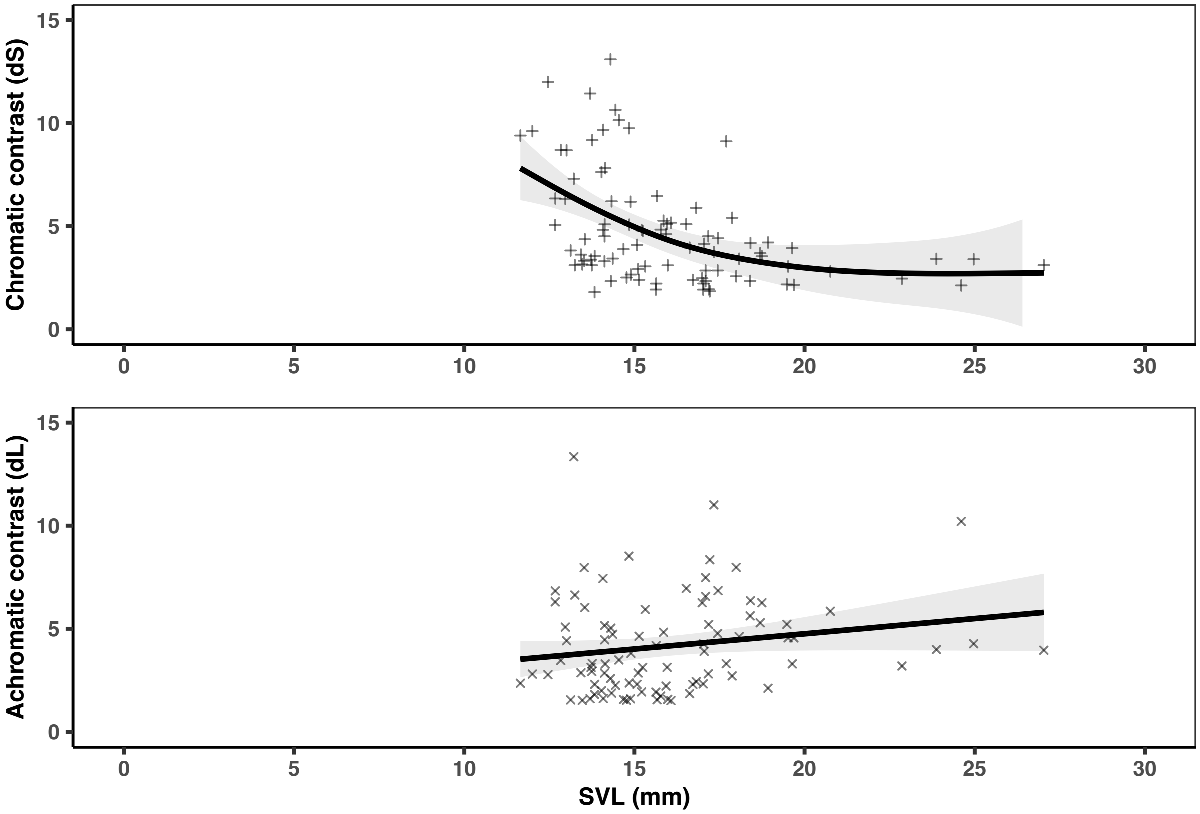


```
# Save plots as Figure 2
jpeg(filename="Fig2_ColourContrast.jpeg",width = 120, height=120, units='mm', bg='white', res=300)
plot(combined_plot)
dev.off()
```

```
## pdf
##   2
```

# Analysis 2 - Behaviour analysis

McEwen BL, Yeager J, Veneat A, & Barnett JB

2025-06-23

## Analysis 2 - Behaviour analysis

Here we will assess how the behaviour of Allobates zaparo varies according to body size & mimicry, when confronted with a novel environment and under two different lighting conditions (high & low light intensity).

In this Rmarkdown script we will analyse: 1. How the probability of entering the novel arena varies by body size, colour, & light intensity - Entry Probability 2. How the time taken to enter the novel arena varies by body size, colour, & light intensity - Entry Latency 3. How frog movement through the novel arena varies according to body size, colour, & light intensity - Activity 3a = number of unique squares the frog moves through on the arena floor 3b = number of jumps performed on the arena floor

We have 90 individuals of Al. zaparo (of various ages/sizes) Each Al. zaparo was presented with the novel environment multiple times

Variables included in this analysis:

FocalFrog = unique alphanumeric code assigned to each individual Al. zaparo

vids = unique alphanumeric code assigned to each individual video file

Batch = batch of frogs collected and tested on the same days

Day = day of testing (1 or 2), nested within Batch

Round = round of testing (1 or 2), nested within Day

TimeOfDay = time block when the experiment was performed (morning or afternoon)

LightTmnt = lighting condition (dark = low light; light = high light)

SVL = the length of the frog in mm

entry = binary data on whether the frog enter the arena (1 = entered)

EntryLatency = time taken to enter the arena

SPMT = number of unique floor squares entered per minute on the arena floor

JPMT = activity - number of jumps performed per minute on the arena floor

DorsalColJND = chromatic contrast (dS) between focal Al. zaparo & Am. bilinguis

DorsalLumJND = achromatic contrast (dL) between focal Al. zaparo & Am. bilinguis

```
# First, loading the required packages:
library(tidyverse) # Organizing the data
```

```
## -- Attaching core tidyverse packages ------------------------ tidyverse 2.0.0 --
## v dplyr     1.1.4     v readr     2.1.5
## v forcats   1.0.0     v stringr   1.5.1
## v ggplot2   3.5.2     v tibble    3.3.0
## v lubridate 1.9.4     v tidyr     1.3.1
```

```
## v purrr     1.0.4
## -- Conflicts ------------------------------------------ tidyverse_conflicts() --
## x dplyr::filter() masks stats::filter()
## x dplyr::lag()    masks stats::lag()
## i Use the conflicted package (<http://conflicted.r-lib.org/>) to force all conflicts to become errors
```

```
library(glmmTMB) # Performing the analysis
library(car) # Comparing between models
```

```
## Loading required package: carData
##
## Attaching package: 'car'
##
## The following object is masked from 'package:dplyr':
##
##     recode
##
## The following object is masked from 'package:purrr':
##
##     some
```

```
library(emmeans) # Calculating pariwise contrasts
```

```
## Welcome to emmeans.
## Caution: You lose important information if you filter this package's results.
## See '? untidy'
```

```
library(DHARMa) # Checking model fit / assumptions
```

```
## This is DHARMa 0.4.7. For overview type '?DHARMa'. For recent changes, type news(package = 'DHARMa')
```

```
library(performance) # Checking model fit / assumptions
library(ggplot2) # Plotting the data
library(patchwork) # Combining plots into a multifaceted figure

# Clear the work space
rm(list=ls())
```

### Read in and check the data

```
# Read in all of the behaviour & colour contrast data
m1dat <- read.csv("MS_BehaviourData.csv", stringsAsFactors = T)

m1dat$Day <- as.factor(m1dat$Day) # make Day a factor variable
m1dat$TimeOfDay <- factor(m1dat$TimeOfDay, # Make time of day an ordered factor
                        levels=c("Morning", "Afternoon"))
```

### Analysing Entry Probability

```
m1 <- glmmTMB(entry ~ LightTmnt + TimeOfDay + SVL +
                DorsalColJND + DorsalLumJND +
                (1|Batch/Day/Round) + (1|FocalFrog),
              data = m1dat,
              family="binomial")
```

```
# Plot the residuals and check model fit
simulateResiduals(fittedModel = m1, plot=T)
```

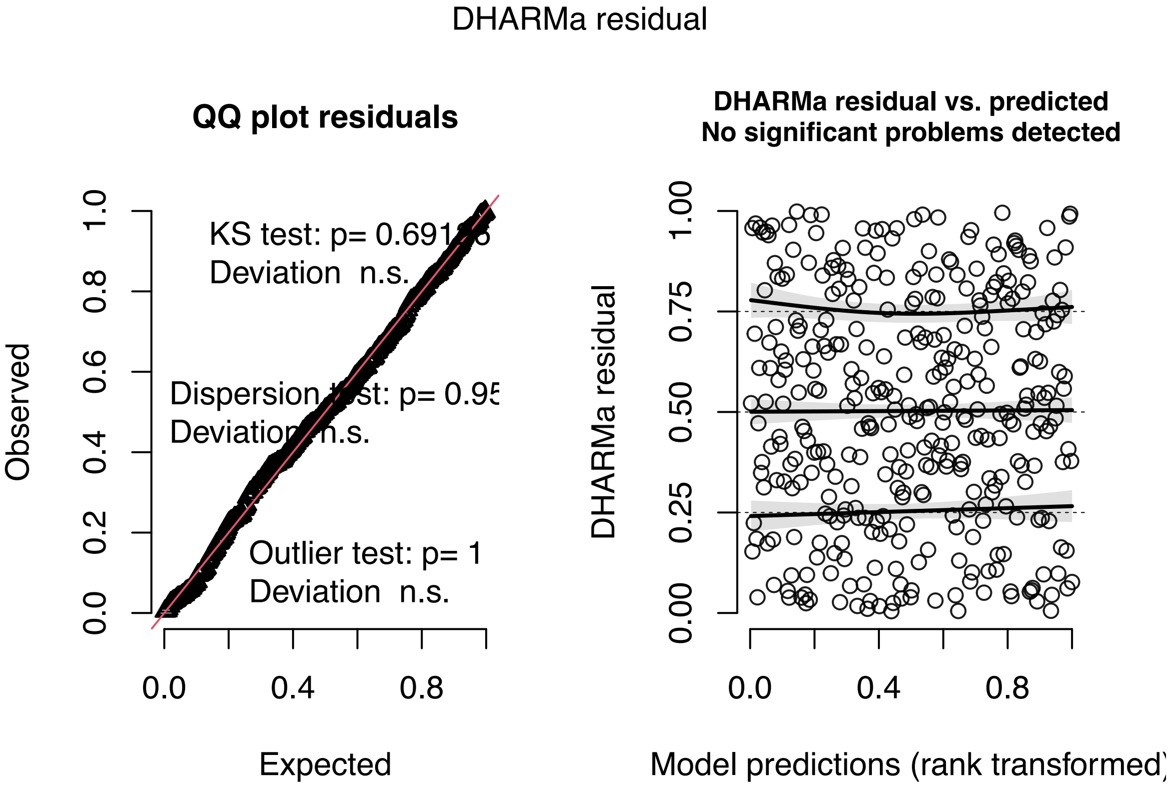


```
## Object of Class DHARMa with simulated residuals based on 250 simulations with refit = FALSE . See ?DI
##
## Scaled residual values: 0.8228018 0.6050306 0.1077925 0.2422696 0.2314628 0.66406 0.5461957 0.036084
```

```
# Check for any collinearity in the model
plot(check_collinearity(m1))
```

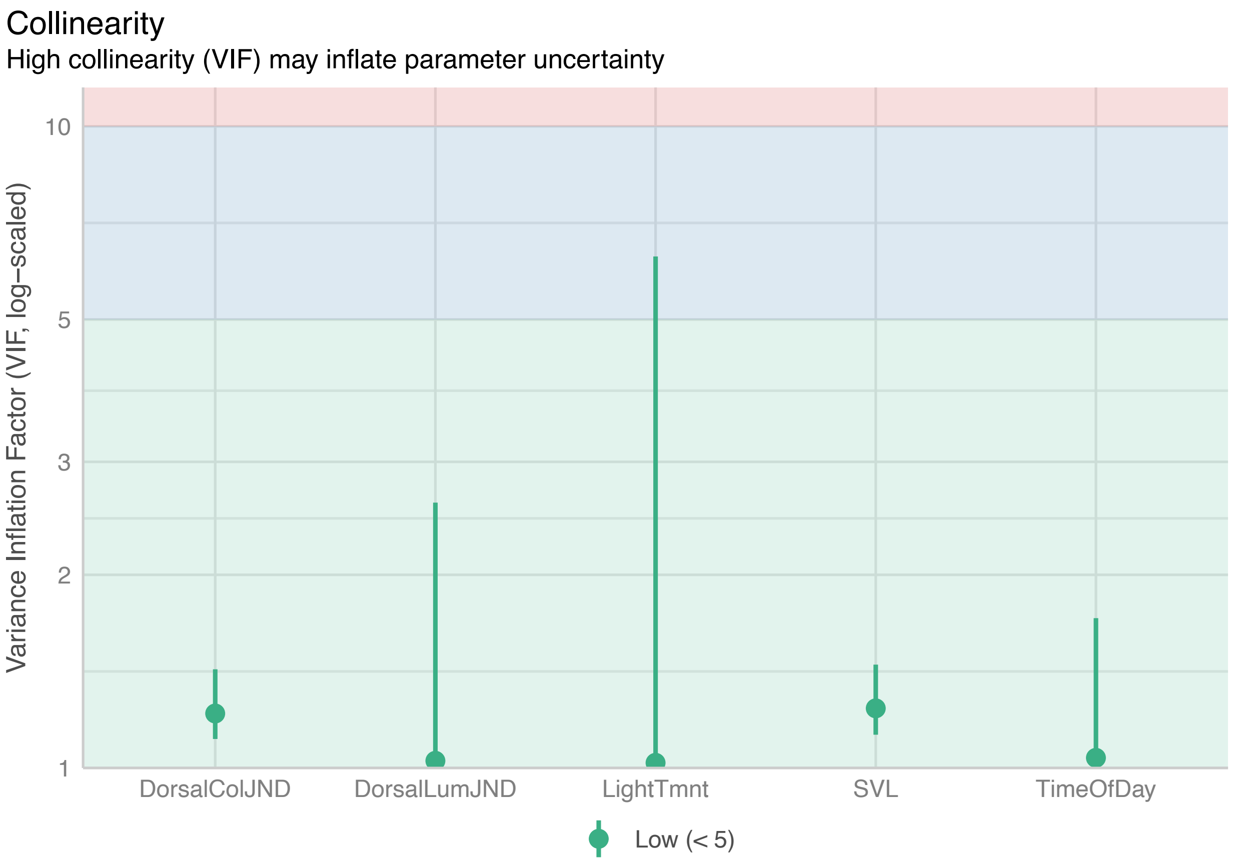


```r
# All tests are non-significant (p > 0.05)
# We can reasonably conclude that the model fits the data
# and conforms to all assumptions.

# Report the main effects from the model
Anova(m1, type="III")
```

```
## Analysis of Deviance Table (Type III Wald chisquare tests)
##
## Response: entry
##                Chisq Df Pr(>Chisq)
## (Intercept)  15.7374  1  7.277e-05 ***
## LightTmnt     2.9565  1  0.0855320 .
## TimeOfDay    11.3047  1  0.0007731 ***
## SVL           8.3744  1  0.0038053 **
## DorsalColJND  0.0026  1  0.9591372
## DorsalLumJND  0.5137  1  0.4735430
## ---
## Signif. codes:  0 '***' 0.001 '**' 0.01 '*' 0.05 '.' 0.1 ' ' 1
```

```r
# There is a significant relationship between Entry Probability & body size
# (entry ~ SVL: X2 = 8.37, df = 1, p = 0.004)
# And between Entry Probability & time of day
# (entry ~ TimeOfDay: X2 = 11.31, df = 1, p < 0.001)
# All other variables are non-significant (p > 0.05)

# As both size and time of day have a significant effect
```

```r
# we will check whether there is an interaction between these two variables
m1.posthocinteraction <- glmmTMB(entry ~ LightTmnt + TimeOfDay*SVL +
                                   DorsalColJND + DorsalLumJND +
                                   (1|Batch/Day/Round) + (1|FocalFrog),
                                 data=m1dat,
                                 family="binomial")
# Compare the original additive model to the interactive model
anova(m1, m1.posthocinteraction)
```

```
## Data: m1dat
## Models:
## m1: entry ~ LightTmnt + TimeOfDay + SVL + DorsalColJND + DorsalLumJND + , zi=~0, disp=~1
## m1:     (1 | Batch/Day/Round) + (1 | FocalFrog), zi=~0, disp=~1
## m1.posthocinteraction: entry ~ LightTmnt + TimeOfDay * SVL + DorsalColJND + DorsalLumJND + , zi=~0, d
## m1.posthocinteraction:     (1 | Batch/Day/Round) + (1 | FocalFrog), zi=~0, disp=~1
##                         Df    AIC    BIC  logLik deviance  Chisq Chi Df
## m1                      10 279.11 317.72 -129.55   259.11
## m1.posthocinteraction 11 281.06 323.53 -129.53   259.06 0.0429      1
##                         Pr(>Chisq)
## m1
## m1.posthocinteraction     0.8359
```

```r
# The interactive model is not significantly different to the additive model
# (X2 = 0.04, df = 1, p = 0.836)
```

### Analysing Entry Latency

```r
# Frogs that did not enter have latency of NA and are excluded from the model
m2 <- glmmTMB(EntryLatency ~ LightTmnt + SVL + TimeOfDay +
                DorsalColJND + DorsalLumJND +
                (1|Batch/Day/Round) + (1|FocalFrog),
              data=m1dat, family=Gamma(link="log"))

# Plot the residuals and check model fit
simulateResiduals(fittedModel=m2, plot=T)
```

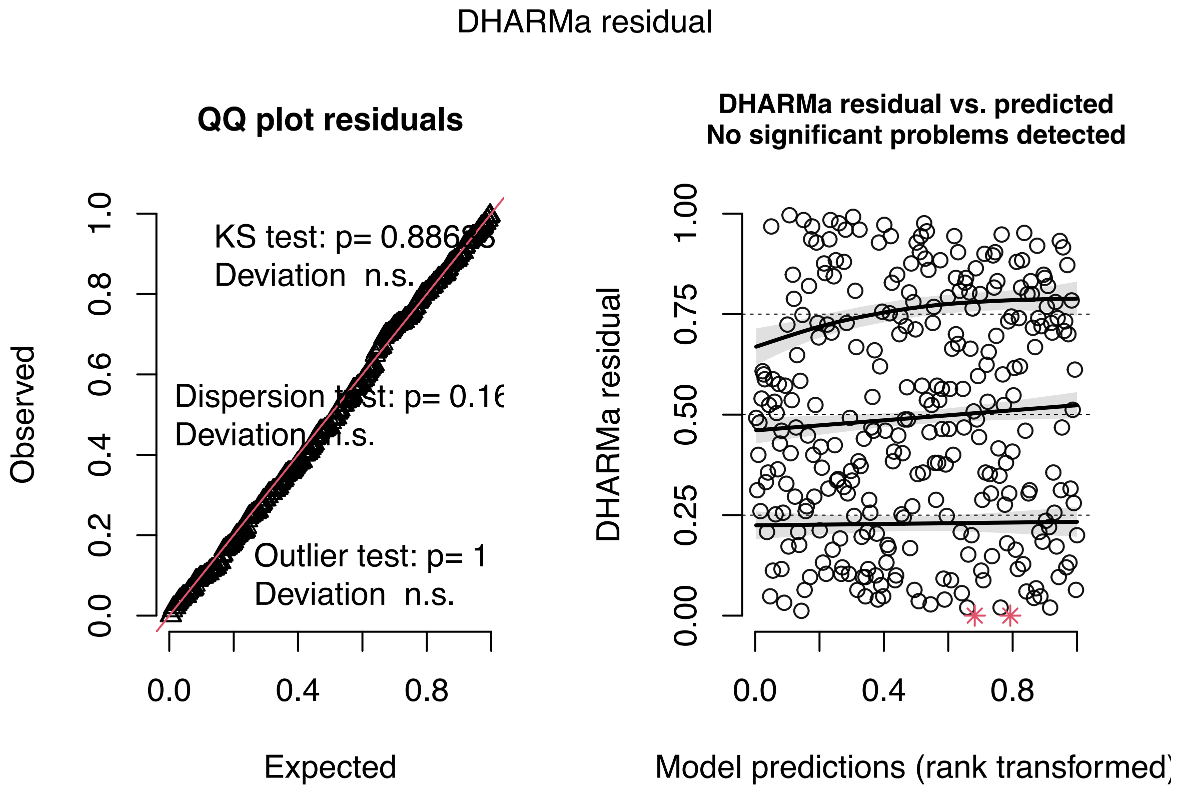


```
## Object of Class DHARMa with simulated residuals based on 250 simulations with refit = FALSE . See ?DH
##
## Scaled residual values: 0.048 0.068 0.096 0.74 0.112 0.804 0.588 0.88 0.46 0.32 0.504 0.54 0.404 0.8
```

```
# Check for any collinearity in the model
plot(check_collinearity(m2))
```

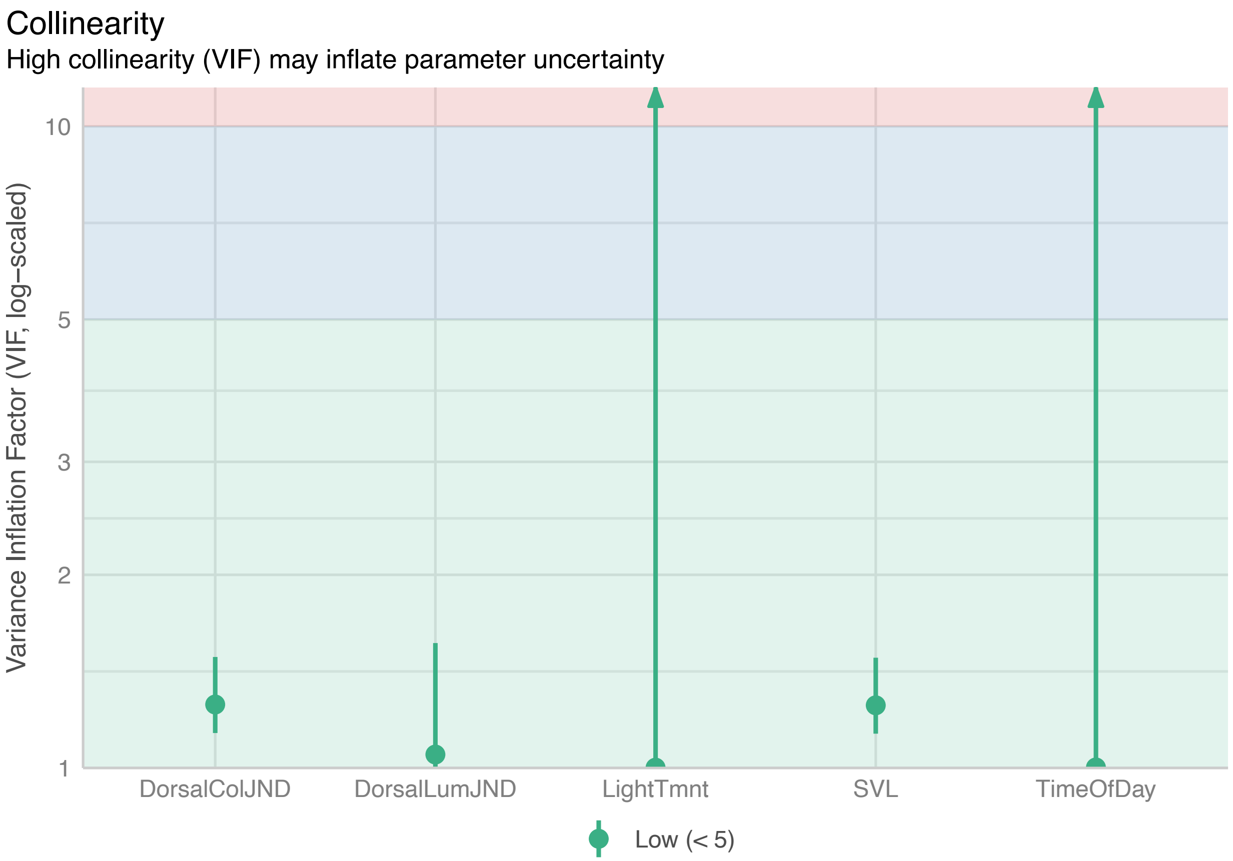


```r
# All tests are non-significant (p > 0.05)
# We can reasonably conclude that the model fits the data
# and conforms to all assumptions.

# Report the main effects from the model
Anova(m2, type="III")
```

```
## Analysis of Deviance Table (Type III Wald chisquare tests)
##
## Response: EntryLatency
##                 Chisq Df Pr(>Chisq)
## (Intercept)   52.1767  1  5.072e-13 ***
## LightTmnt      0.7226  1  0.3952826
## SVL            4.4281  1  0.0353508 *
## TimeOfDay     13.2135  1  0.0002779 ***
## DorsalColJND   0.6894  1  0.4063655
## DorsalLumJND   0.5564  1  0.4556979
## ---
## Signif. codes:  0 '***' 0.001 '**' 0.01 '*' 0.05 '.' 0.1 ' ' 1
```

```r
# There is a significant relationship between Entry Latency & body size
# EntryLatency ~ SVL: X2 = 4.43, df = 1, p = 0.035)
# And between Entry Latency & time of day
# (EntryLatency ~ TimeOfDay: X2 = 13.21, df = 1, p < 0.001)
# All other variables are non-significant (p > 0.05)
```

```r
# As both size and time of day have a significant effect
# we will check whether there is an interaction between these two variables
m2.posthocinteraction <- glmmTMB(EntryLatency ~ LightTmnt + SVL*TimeOfDay +
                                   DorsalColJND + DorsalLumJND +
                                   (1|Batch/Day/Round) + (1|FocalFrog),
                                 data=m1dat, family=Gamma(link="log"))
# Compare the original additive model to the interactive model
anova(m2, m2.posthocinteraction)
```

```
## Data: m1dat
## Models:
## m2: EntryLatency ~ LightTmnt + SVL + TimeOfDay + DorsalColJND + DorsalLumJND + , zi=~0, disp=~1
## m2:     (1 | Batch/Day/Round) + (1 | FocalFrog), zi=~0, disp=~1
## m2.posthocinteraction: EntryLatency ~ LightTmnt + SVL * TimeOfDay + DorsalColJND + DorsalLumJND + , [illegible]
## m2.posthocinteraction:     (1 | Batch/Day/Round) + (1 | FocalFrog), zi=~0, disp=~1
##                       Df    AIC    BIC  logLik deviance  Chisq Chi Df
## m2                    11 3779.7 3820.4 -1878.8   3757.7
## m2.posthocinteraction 12 3780.0 3824.4 -1878.0   3756.0 1.6837      1
##                       Pr(>Chisq)
## m2
## m2.posthocinteraction     0.1944
```

```r
# The interactive model is not significantly different to the additive model
# (X2 = 1.68, df = 1, p = 0.194)
```

**Analysing activity (number of squares)**

**We will analyse activity in two ways (focusing on terrestrial movement)**

1. **The number of unique squares crossed**
2. **The number of jumps performed**

**As frogs spent different amounts of time on the arena floor**

**we will analyse activity per unit time (minute)**

**SPMT = squares per minute**

**JPMT = jumps per minute**

```r
# Squares crossed per minute (terrestrial)
hist(m1dat$SPMT) # Check the distribution of raw data
```

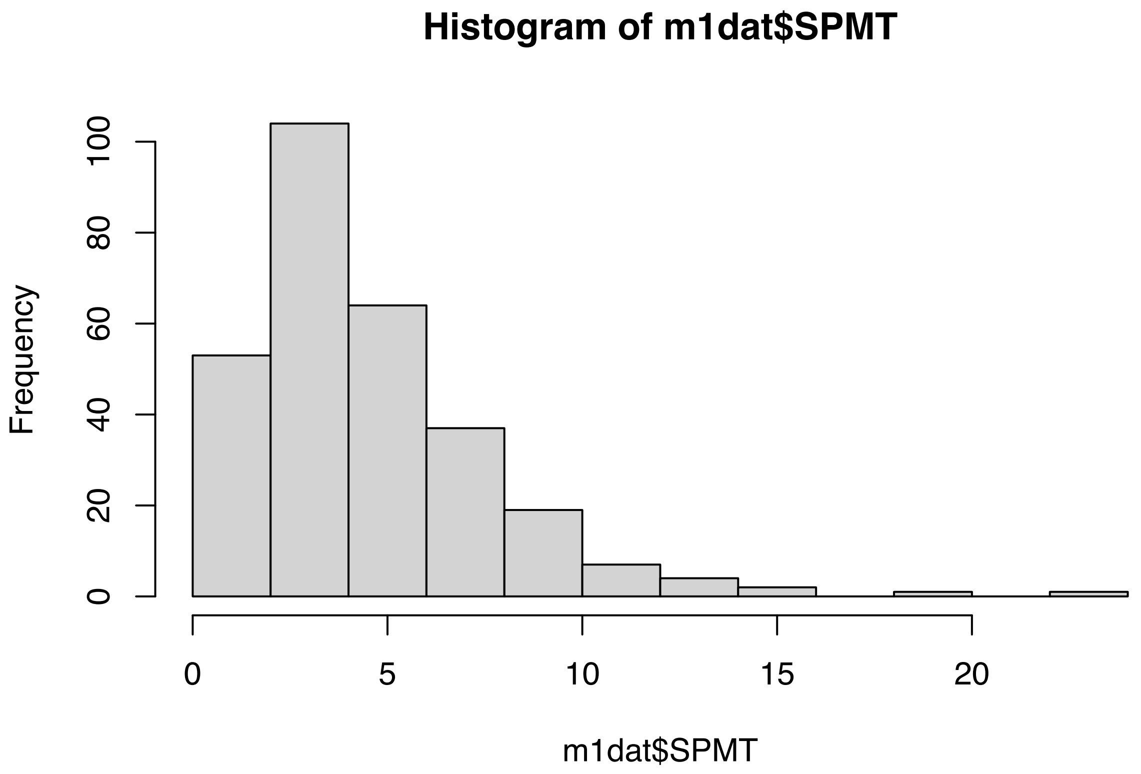


```
hist(log(m1dat$SPMT)) # Check the distribution of log transformed data
```

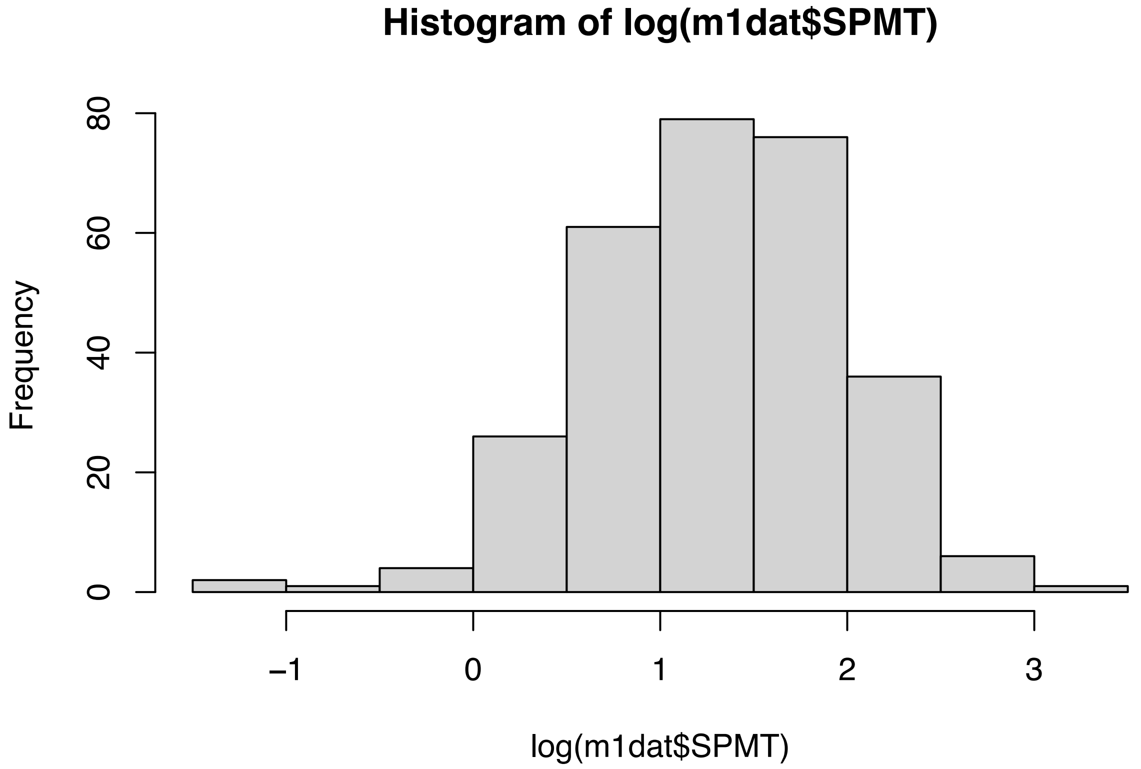


```
# log transformed data approximates a normal distribution
# analyse log transformed SPMT data with a Gaussian error distribution
m3 <- glmmTMB(log(SPMT) ~ LightTmnt + TimeOfDay + SVL +
```

```
                DorsalColJND + DorsalLumJND +
                (1|Batch/Day/Round) + (1|FocalFrog),
              family="gaussian",
              data=m1dat)
# Check the residuals
simulateResiduals(fittedModel=m3, plot=T)
```

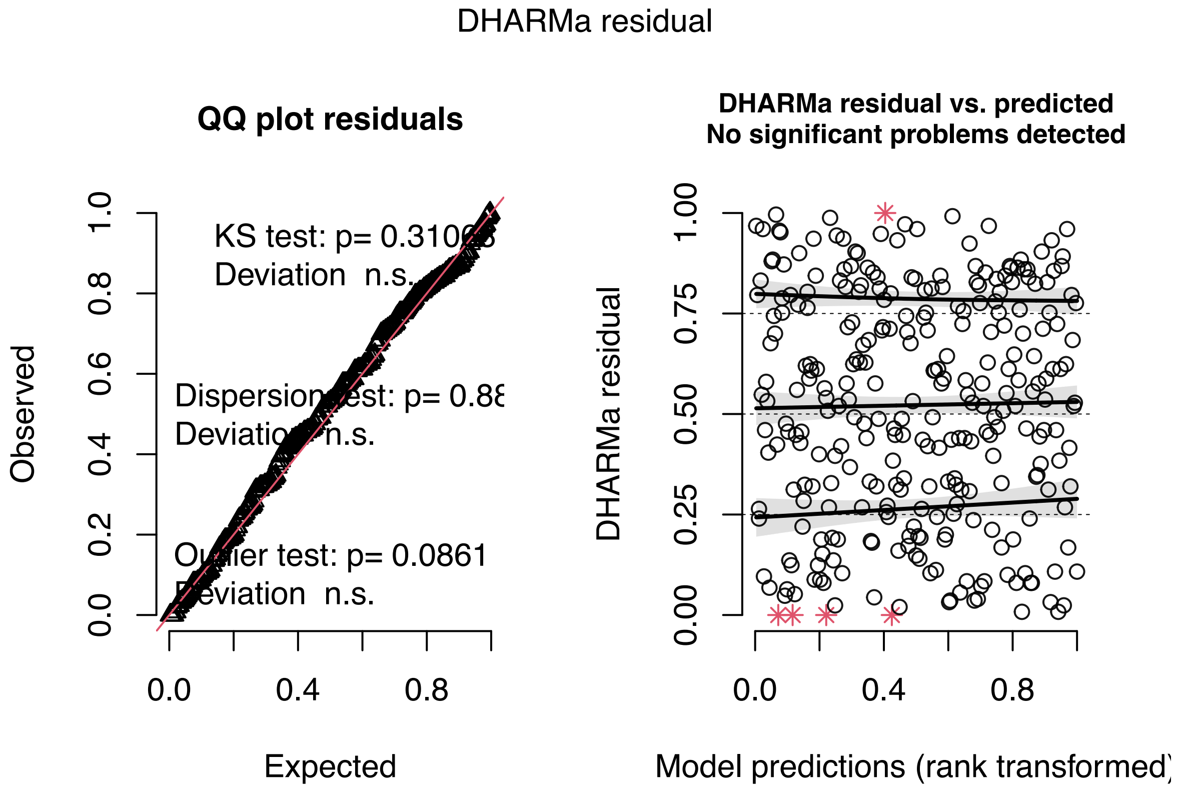


```
## Object of Class DHARMa with simulated residuals based on 250 simulations with refit = FALSE . See ?DH
##
## Scaled residual values: 0.764 0.712 0.804 1 0.24 0.628 0.264 0.788 0.584 0.752 0.416 0.88 0.528 0.71
```

```
# In all tests p > 0.05, the data conforms to the model assumptions
# Check for any collinearity in the model
plot(check_collinearity(m3))
```

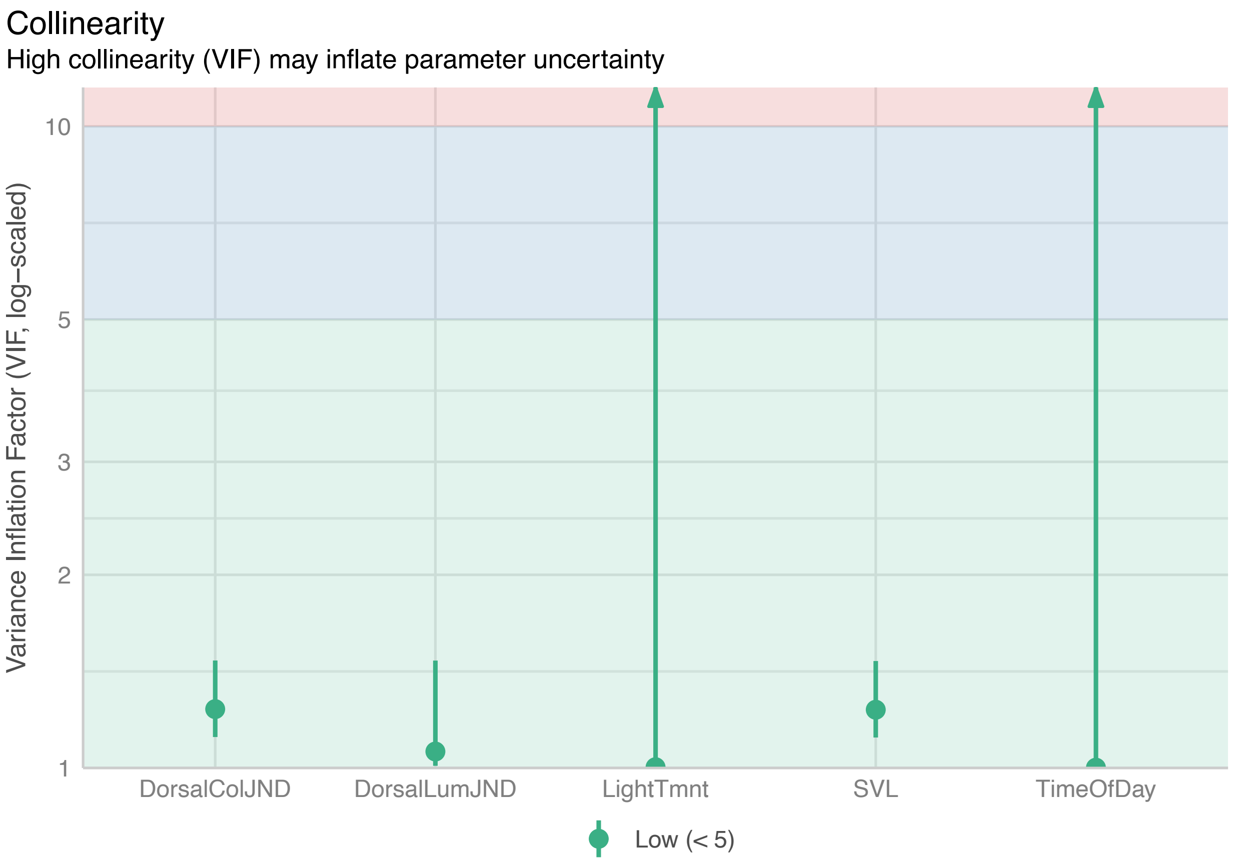


```r
# There is no significant collinearity

# Summarize the results from the model
Anova(m3, type="III")
```

```
## Analysis of Deviance Table (Type III Wald chisquare tests)
##
## Response: log(SPMT)
##                 Chisq Df Pr(>Chisq)
## (Intercept)    7.6148  1  0.0057890 **
## LightTmnt     14.2619  1  0.0001591 ***
## TimeOfDay      1.6222  1  0.2027853
## SVL            0.4022  1  0.5259627
## DorsalColJND   0.8745  1  0.3497250
## DorsalLumJND   0.0110  1  0.9163837
## ---
## Signif. codes:  0 '***' 0.001 '**' 0.01 '*' 0.05 '.' 0.1 ' ' 1
```

```r
# There is a significant effect of light treatment (X2=14.26, df=1, p<0.001)
# But all other variables are non-significant (p>0.05)
```

```r
# Jumps per minute (terrestrial)
hist(m1dat$JPMT) # Check the distribution of raw data
```

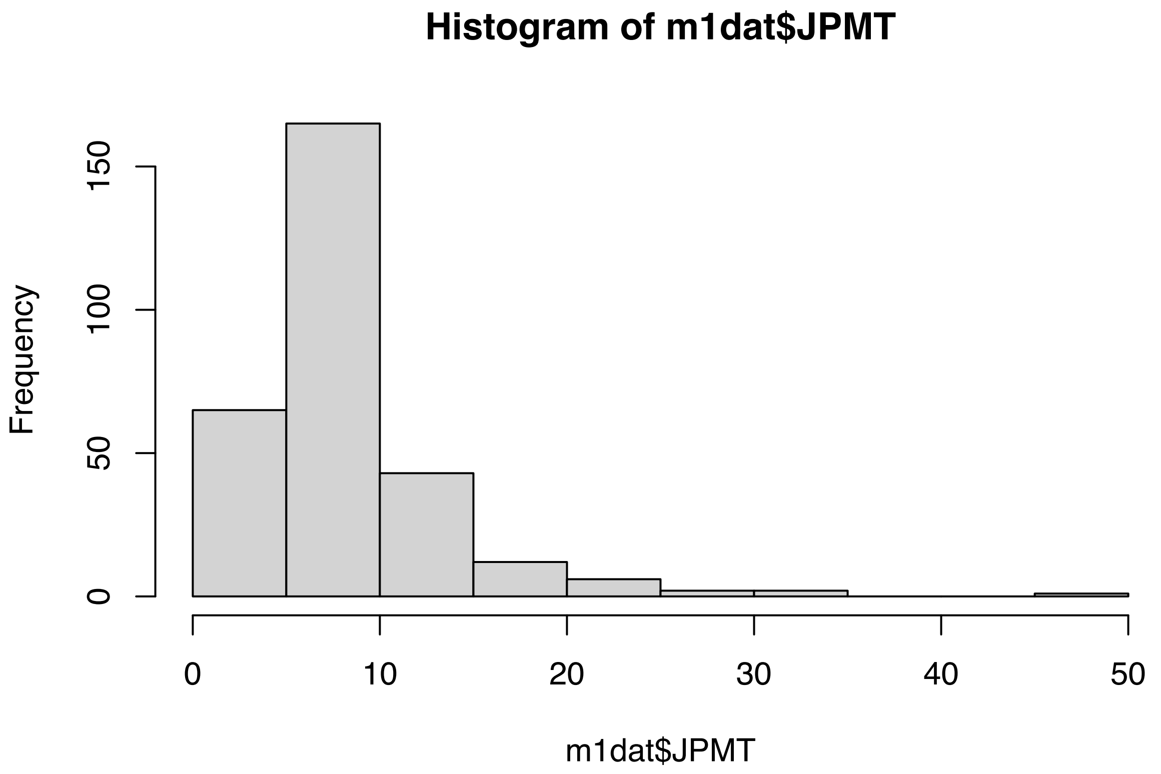


```
hist(log(m1dat$JPMT)) # Check the distribution of log transformed data
```

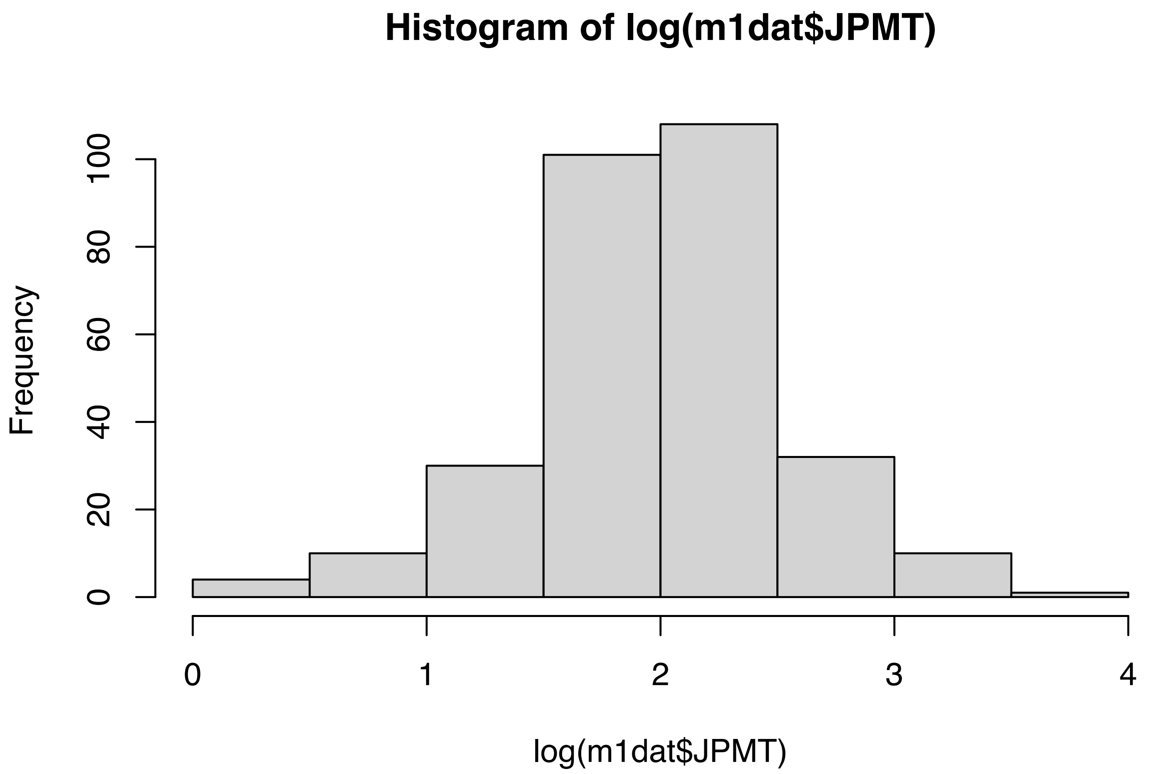


```
# log transformed data approximates a normal distribution
# analyse log transformed SPMT data with a Gaussian error distribution
m4 <- glmmTMB(log(JPMT) ~ LightTmnt + SVL + TimeOfDay +
```

```
                 DorsalColJND + DorsalLumJND +
                 (1|Batch/Day/Round) + (1|FocalFrog),
               family="gaussian",
               data=m1dat)
# Check the residuals
simulateResiduals(fittedModel=m4, plot=T)
```

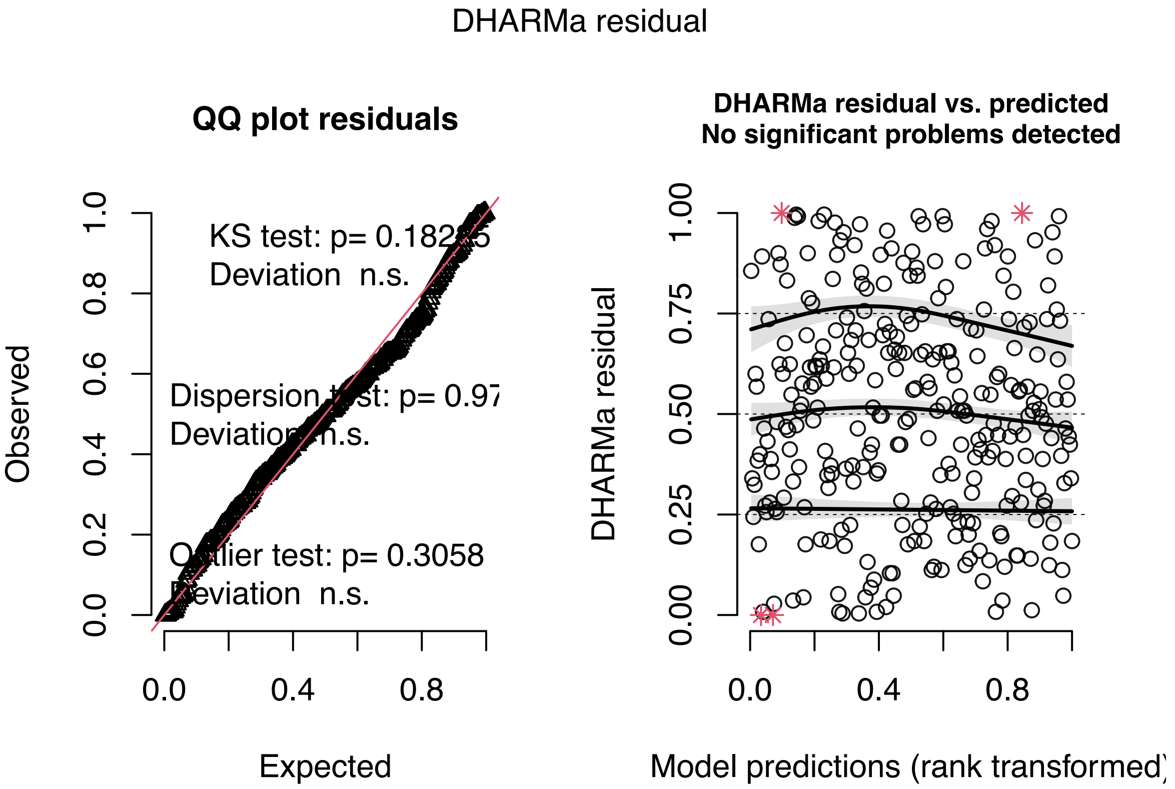


```
## Object of Class DHARMa with simulated residuals based on 250 simulations with refit = FALSE . See ?DI
##
## Scaled residual values: 0.412 0.548 0.624 0.896 0.332 0.036 0.48 0.544 0.256 0.424 0.6 0.372 0.74 0.:
```

```
# In all tests p > 0.05, the data conforms to the model assumptions
# Check for any collinearity in the model
plot(check_collinearity(m4))
```

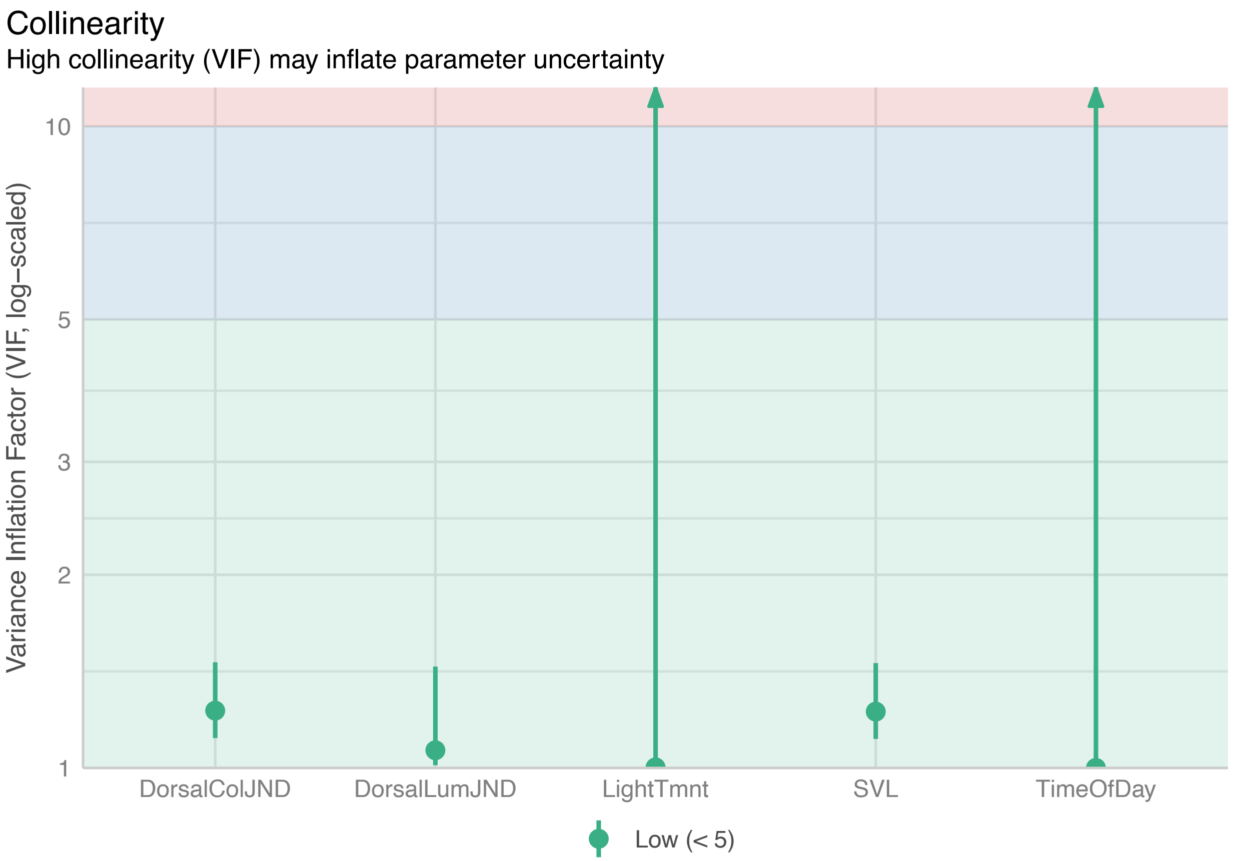


```r
# There is no significant collinearity

# Summarize the results from the model
Anova(m4, type="III")
```

```
## Analysis of Deviance Table (Type III Wald chisquare tests)
##
## Response: log(JPMT)
##                Chisq Df Pr(>Chisq)
## (Intercept)  74.5705  1    < 2e-16 ***
## LightTmnt     5.3203  1    0.02108 *
## SVL           2.7257  1    0.09874 .
## TimeOfDay     5.0734  1    0.02430 *
## DorsalColJND  2.5403  1    0.11097
## DorsalLumJND  0.5585  1    0.45485
## ---
## Signif. codes:  0 '***' 0.001 '**' 0.01 '*' 0.05 '.' 0.1 ' ' 1
```

```r
# There is a significant effect of light treatment (X2=5.32, df=1, p=0.021)
# And a significant effect of time of day (X2=5.07, df=1, p=0.024)
# All other variables are non-significant (p>0.05)

# As there are two variables significantly affecting the number of jumps
# we will check for an interaction between light treatment & time of day

m4.posthocinteraction <- glmmTMB(log(JPMT) ~ LightTmnt*TimeOfDay + SVL +
                                   DorsalColJND + DorsalLumJND +
```

```
                                   (1|Batch/Day/Round) + (1|FocalFrog),
                                 family="gaussian",
                                 data=m1dat)
anova(m4, m4.posthocinteraction)
```

```
## Data: m1dat
## Models:
## m4: log(JPMT) ~ LightTmnt + SVL + TimeOfDay + DorsalColJND + DorsalLumJND + , zi=~0, disp=~1
## m4:     (1 | Batch/Day/Round) + (1 | FocalFrog), zi=~0, disp=~1
## m4.posthocinteraction: log(JPMT) ~ LightTmnt * TimeOfDay + SVL + DorsalColJND + DorsalLumJND + , zi=
## m4.posthocinteraction:     (1 | Batch/Day/Round) + (1 | FocalFrog), zi=~0, disp=~1
##                       Df    AIC    BIC  logLik deviance  Chisq Chi Df
## m4                    11 461.78 502.37 -219.89   439.78
## m4.posthocinteraction 12 463.52 507.80 -219.76   439.52 0.2611      1
##                       Pr(>Chisq)
## m4
## m4.posthocinteraction     0.6094
```

```
# The interaction term is non-significant (X2 = 0.26, df = 1, p = 0.609)
# So we can interpret the effects of light treatment & time of day separately
```

### Plotting the data

```
# Data for Probability & Latency Plots
EntryData <- m1dat
EntryData$TimeOfDay <- factor(EntryData$TimeOfDay,
                              levels = c("Morning", "Afternoon"))

# Data for Activity plots
# Data for Squares Crossed per Minute Plot
# Calculate the means +/- 95% CI from the model for each light treatment
m3emm <- emmeans(m3, "LightTmnt", type="response")
SquaresPairwiseData <- as.data.frame(m3emm)
# Set treatment level order as Dark (low light) first, then Light (high light)
SquaresPairwiseData$LightTmnt <- factor(SquaresPairwiseData$LightTmnt,
                                        levels = c("Dark", "Light"))

# Data for Jumps per Minute Plot
# Calculate the means +/- 95% CI from the model for
# each time of day within each light treatment
# And each
m4emm <- emmeans(m4, ~ LightTmnt * TimeOfDay)
JumpPairwiseData <- as.data.frame(m4emm)
# Take the exponent of the mean, SE, & 95% CI
# to transform data back into jumps per minute
JumpPairwiseData[,c(3:4,6:7)]<- exp(JumpPairwiseData[,c(3:4,6:7)])

# Set light treatment level order as Dark (low) first, then Light (high)
JumpPairwiseData$LightTmnt <- factor(JumpPairwiseData$LightTmnt,
                                     levels = c("Dark", "Light"))
# Set time of day order as Morning first, then Afternoon
JumpPairwiseData$TimeOfDay <- factor(JumpPairwiseData$TimeOfDay,
                                     levels = c("Morning", "Afternoon"))
```

```r
# Plot the data using ggplot2
##########################
# PROBABILITY PLOT
EntryProbPlot <-
  ggplot(data = EntryData,
         aes(x = SVL, y = entry, color = TimeOfDay)) +
  scale_color_manual(values = c("Morning" = "grey50",
                                "Afternoon" = "black"),
                     name = "Time of Day") +

  geom_point(alpha = 0.5,
             shape = 20,
             size = 1,
             position = position_jitter(height = 0.005)) +
  labs(x = "SVL (mm)",
       y = "Entry Probability") +

  scale_x_continuous(limits = c(0, 30), breaks = seq(0, 30, 5)) +
  scale_y_continuous(limits = c(-0.05, 1.05), breaks = seq(0, 1, 0.2)) +

  scale_fill_discrete(breaks=c('Morning', 'Afternoon')) +

  geom_smooth(method = "glm", method.args=list(family = "binomial"), se = T,
              alpha = 0.5, size = 1) +

  theme_bw() +
  theme(axis.line = element_line(colour = "black"),
        panel.grid.major = element_blank(),
        panel.grid.minor = element_blank(),
        panel.background = element_blank()) +
  theme(panel.grid = element_blank()) +
  theme(panel.grid = element_blank()) +
  theme(plot.title = element_blank()) +

  theme(axis.title.y = element_text(size = 9, face = "bold")) +
  theme(axis.title.x = element_text(size = 9, face = "bold")) +
  theme(axis.text.x = element_text(size = 8, face = "bold")) +
  theme(axis.text.y = element_text(size = 8, face = "bold")) +
  theme(axis.ticks.y = element_line(linewidth = 0.7)) +
  theme(axis.ticks.x = element_line(linewidth = 0.7)) +

  theme(legend.position = c(0.12, 0.8)) +
  theme(legend.title = element_text(size = 6, face = "bold")) +
  theme(legend.text = element_text(size = 6, face = "bold")) +
  theme(legend.key = element_rect(color = NA, fill = NA),
        legend.key.size = unit(0.25, "cm"))
```

```
## Warning: Using `size` aesthetic for lines was deprecated in ggplot2 3.4.0.
## i Please use `linewidth` instead.
## This warning is displayed once every 8 hours.
## Call `lifecycle::last_lifecycle_warnings()` to see where this warning was
## generated.

## Warning: A numeric `legend.position` argument in `theme()` was deprecated in ggplot2
```

```
## 3.5.0.
## i Please use the `legend.position.inside` argument of `theme()` instead.
## This warning is displayed once every 8 hours.
## Call `lifecycle::last_lifecycle_warnings()` to see where this warning was
## generated.
```

```
#########################
# LATENCY PLOT
EntryLatencyPlot <-
  ggplot(data = EntryData,
         aes(x = SVL, y = EntryLatency, group = TimeOfDay, colour = TimeOfDay)) +
  geom_point(aes(fill = TimeOfDay),
             alpha = 0.5,
             shape = 20,
             size = 1) +

  scale_color_manual(values = c("Morning" = "grey50",
                                "Afternoon" = "black"),
                     name = "Time of Day") +

  geom_smooth(method = "lm", se = T) +
  labs(x = "SVL (mm)",
       y = "Entry latency (s)") +

  scale_x_continuous(limits = c(0, 30), breaks = seq(0, 30, 5)) +
  scale_y_continuous(limits = c(0, 1000), breaks = seq(0, 1000, 200)) +

  scale_fill_discrete(breaks = c('Morning', 'Afternoon')) +

  geom_smooth(method = "glm", method.args = list(family = "binomial"), se = T,
              alpha = 0.5, size = 1) +

  theme_bw() +
  theme(axis.line = element_line(colour = "black"),
        panel.grid.major = element_blank(),
        panel.grid.minor = element_blank(),
        panel.background = element_blank()) +
  theme(panel.grid = element_blank()) +
  theme(panel.grid = element_blank()) +
  theme(plot.title = element_blank()) +

  theme(axis.title.y = element_text(size = 9, face = "bold")) +
  theme(axis.title.x = element_text(size = 9, face = "bold")) +
  theme(axis.text.x = element_text(size = 8, face = "bold")) +
  theme(axis.text.y = element_text(size = 8, face = "bold")) +
  theme(axis.ticks.y = element_line(linewidth = 0.7)) +
  theme(axis.ticks.x = element_line(linewidth = 0.7)) +

  guides(fill = "none") +
  guides(group = "none") +

  theme(legend.position = c(0.12, 0.8)) +
  theme(legend.title = element_text(size = 6, face = "bold")) +
  theme(legend.text = element_text(size = 6, face = "bold")) +
```

```r
  theme(legend.key = element_rect(color = NA, fill = NA),
        legend.key.size = unit(0.25, "cm"))

#########################
# SQUARES PER MINUTE PLOT
SquaresMeansPlot <-
  ggplot(data = SquaresPairwiseData,
         aes(x = LightTmnt, y = response, color = LightTmnt)) +
  geom_point(size = 2) +

  geom_errorbar(aes(ymin = lower.CL, ymax = upper.CL), size = 1, width = 0) +

  scale_y_continuous(limits = c(0, 10), breaks = seq(0, 10, 2)) +

  scale_x_discrete(labels = c('Low', 'High')) +

  scale_color_manual(
    values = c("Light" = "black", "Dark" = "black"),
    name = "Treatment") +

  labs(x = "Lighting",
       y = "Activity (squares/min)") +

  theme_bw() +
  theme(axis.line = element_line(colour = "black"),
        panel.grid.major = element_blank(),
        panel.grid.minor = element_blank(),
        panel.background = element_blank()) +
  theme(panel.grid = element_blank()) +
  theme(panel.grid = element_blank()) +
  theme(plot.title = element_blank()) +

  theme(axis.title.y = element_text(size = 9, face = "bold")) +
  theme(axis.title.x = element_text(size = 9, face = "bold")) +
  theme(axis.text.x = element_text(size = 8, face = "bold")) +
  theme(axis.text.y = element_text(size = 8, face = "bold")) +
  theme(axis.ticks.y = element_line(linewidth = 0.7)) +
  theme(axis.ticks.x = element_line(linewidth = 0.7)) +

  theme(legend.position="none")

#########################
# JUMPS PER MINUTE PLOT
JumpMeansPlot <-
  ggplot(data = JumpPairwiseData,
         aes(x = LightTmnt, y = emmean, group = TimeOfDay,
             color = TimeOfDay)) +
  geom_point(size = 2,position=position_dodge(width=0.5)) +


  geom_errorbar(aes(ymin = lower.CL, ymax = upper.CL), size = 1, width = 0,
                position=position_dodge(width=0.5)) +
```

```
  scale_y_continuous(limits = c(0, 10), breaks = seq(0, 10, 2)) +

  scale_x_discrete(labels = c('Low', 'High')) +

  scale_color_manual(values = c("Morning" = "grey50",
                                "Afternoon" = "black"),
                     name = "Time of Day") +

  labs(x = "Lighting",
       y = "Activity (jumps/min)") +

  theme_bw() +
  theme(axis.line = element_line(colour = "black"),
        panel.grid.major = element_blank(),
        panel.grid.minor = element_blank(),
        panel.background = element_blank()) +
  theme(panel.grid = element_blank()) +
  theme(panel.grid = element_blank()) +
  theme(plot.title = element_blank()) +

  theme(axis.title.y = element_text(size = 9, face = "bold")) +
  theme(axis.title.x = element_text(size = 9, face = "bold")) +
  theme(axis.text.x = element_text(size = 8, face = "bold")) +
  theme(axis.text.y = element_text(size = 8, face = "bold")) +
  theme(axis.ticks.y = element_line(linewidth = 0.7)) +
  theme(axis.ticks.x = element_line(linewidth = 0.7)) +


  guides(fill = "none") +
  guides(group = "none") +

  theme(legend.position = c(0.25, 0.2)) +
  theme(legend.title = element_text(size = 6, face = "bold")) +
  theme(legend.text = element_text(size = 6, face = "bold")) +
  theme(legend.key = element_rect(color = NA, fill = NA),
        legend.key.size = unit(0.25, "cm"))


# Combining the plots together into one multifaceted figure
layout <- "AA
           BB
           CD"

combined_plot_behaviour2 <- EntryProbPlot + EntryLatencyPlot + SquaresMeansPlot + JumpMeansPlot +
  plot_layout(design = layout)
print(combined_plot_behaviour2)
```

```
## `geom_smooth()` using formula = 'y ~ x'
## `geom_smooth()` using formula = 'y ~ x'

## Warning: Removed 52 rows containing non-finite outside the scale range
## (`stat_smooth()`).

## `geom_smooth()` using formula = 'y ~ x'
```

```
## Warning: Removed 52 rows containing non-finite outside the scale range
## (`stat_smooth()`).

## Warning: Failed to fit group 1.
## Caused by error:
## ! y values must be 0 <= y <= 1

## Warning: Failed to fit group 2.
## Caused by error:
## ! y values must be 0 <= y <= 1

## Warning: Removed 52 rows containing missing values or values outside the scale range
## (`geom_point()`).
```

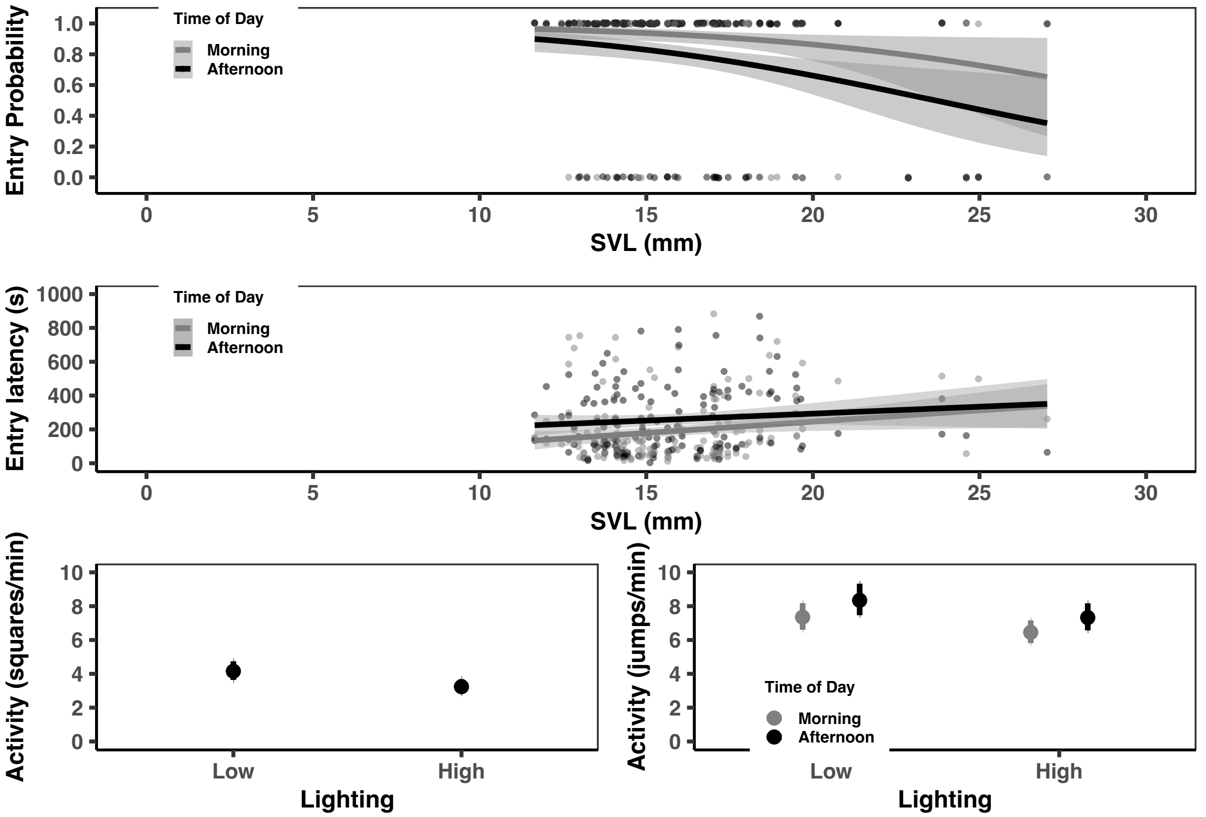


```
# Save plots as Figure 3
jpeg(filename="Fig3_BehaviourAnalysis.jpeg",width = 120, height=180, units='mm', bg='white', res=300)
plot(combined_plot_behaviour2)
```

```
## `geom_smooth()` using formula = 'y ~ x'
## `geom_smooth()` using formula = 'y ~ x'

## Warning: Removed 52 rows containing non-finite outside the scale range
## (`stat_smooth()`).

## `geom_smooth()` using formula = 'y ~ x'

## Warning: Removed 52 rows containing non-finite outside the scale range
## (`stat_smooth()`).

## Warning: Failed to fit group 1.
## Caused by error:
## ! y values must be 0 <= y <= 1
```

```
## Warning: Failed to fit group 2.
## Caused by error:
## ! y values must be 0 <= y <= 1
```

```
## Warning: Removed 52 rows containing missing values or values outside the scale range
## (`geom_point()`).
```

```r
dev.off()
```

```
## pdf
##   2
```

# Analysis 3 - Supplementary Material - Light environment analysis

McEwen BL, Yeager J, Veneat A, & Barnett JB

2025-06-23

## Analysis S1 - Experimental & natural light conditions

Here we will compare the light intensity treatments used in the experiment to measurements taken in the natural habitat of Allobates zaparo

We have two experimental conditions (low & high light intensity) We have three field locations: Forest (F) reserve - 3 sites (F1, F2, & F3) within the close canopy forest Disturbed trail (D) - 5 sites along a trail with a broken canopy Research station (I) - 4 sites, at beginning (dawn) & end (dusk) of activity

Variables included in this analysis:

Habitat = the habitat where measurements were taken - BehaviourArena = the experimental enclosure - Forest = the closed canopy forest - Trail = the broken canopy trail - Iyarina = the research station

Code = unique alphanumeric code assigned to each measurement site

TimeBlock = the time when a measurement was taken - Morning (09:00 - 12:30) - Afternoon (14:30 - 16:00)

Lux = illumination in lux (lx)

```r
# First, loading the required packages:
library(tidyverse) # Organizing the data
```

```
## -- Attaching core tidyverse packages ------------------------ tidyverse 2.0.0 --
## v dplyr     1.1.4     v readr     2.1.5
## v forcats   1.0.0     v stringr   1.5.1
## v ggplot2   3.5.2     v tibble    3.3.0
## v lubridate 1.9.4     v tidyr     1.3.1
## v purrr     1.0.4
## -- Conflicts ------------------------------------------ tidyverse_conflicts() --
## x dplyr::filter() masks stats::filter()
## x dplyr::lag()    masks stats::lag()
## i Use the conflicted package (<http://conflicted.r-lib.org/>) to force all conflicts to become errors
```

```r
library(ggplot2) # Plotting the data
library(patchwork) # Combining plots into a multifaceted figure

# Clear the work space
rm(list=ls())
```

### Read in and check the data

```r
# Read in all of illuminance data
# Including for the experimental arena and the three field locations
LightData <- read.csv("McEwen-etal-2025_LightEnvironment.csv")

# We need to remove data from the road and forest ends of the disturbed trail
```

```r
# As here we are interested in the broken canopy / disturbed part of the habitat
# 'Trail Head' is on the road and is not representative of the frogs' habitat
# 'Just inside forest' is under the full canopy (equivalent to the forest site)
LightData <- LightData[!(LightData$Sub_Site %in% "Trail Head"),]
LightData <- LightData[!(LightData$Sub_Site %in% "Just Inside Forest"),]

# Set Time Block order as Morning first, then Afternoon
LightData$TimeBlock <- factor(LightData$TimeBlock,
                              levels = c("Morning", "Afternoon"))

# Check that we have the correct number of data points
summary(factor(LightData$Code))
```

```
##   D EXP  F1  F2  F3   I
##  25  14  20  20  15   8
```

```r
# There are 25 values for the disturbed trail (D)
#     5 replicates of 5 sites
# There are 14 values for the experimental arena (EXP)
#     7 measurements for each of the high & low light conditions
# There are 20, 20, & 15 measurements for the Forest sites (F1, F2, & F3)
#     4 measurements of 5 sites for F1 & F2 (3 measurements of 5 sites for F3)
# There are 8 measurements for the Research Station (I)
#     2 measurements of 4 sites (2 mornings & 2 evenings)

# This is correct
```

**We can now calculate summary statistics for the experimental treatments**

```r
# Calculate means and SD from the experimental arena
treatment_means <- LightData %>%
  filter(Habitat=="BehaviourArena") %>%
  group_by(Sub_Site)%>%
  summarise(mean_lux = mean(Lux, na.rm=T),
            sd_lux = sd(Lux, na.rm=T))

# Calculate means +/- SD for both experimental conditions
treatment_means <- as.data.frame(treatment_means)
treatment_means$lwr <- treatment_means$mean_lux - treatment_means$sd_lux
treatment_means$upr <- treatment_means$mean_lux + treatment_means$sd_lux

# Display the results
treatment_means
```

```
##         Sub_Site   mean_lux    sd_lux        lwr        upr
## 1 High Treatment 1167.28571 33.732844 1133.55287 1201.01856
## 2  Low Treatment   63.57143  6.754187   56.81724   70.32562
```

**And the summary statistics for each field location**

```r
# Calculate summary statistics for the
# Forest, Disturbed Trail, and the Research Station
# separately for the morning and afternoon time blocks
# mean calculated across the repeated measures
```

```
# of each time block (TimeBlock) within each location (Code)

# Forest (F)
forestdata <-   LightData %>%
  filter(Habitat == "Forest")
aggregate(Lux ~ TimeBlock * Code, data = forestdata, summary)
```

```
##   TimeBlock Code Lux.Min. Lux.1st Qu. Lux.Median Lux.Mean Lux.3rd Qu. Lux.Max.
## 1   Morning   F1   483.00      712.75     980.00   968.80     1244.00  1480.00
## 2 Afternoon   F1   191.00      345.25     570.50   640.10      833.25  1368.00
## 3   Morning   F2   660.00      788.00     859.50  1031.50     1238.00  1810.00
## 4 Afternoon   F2   287.00      537.00     846.00   823.90     1126.00  1258.00
## 5   Morning   F3   630.00      932.00    1136.00  1213.20     1348.00  2020.00
## 6 Afternoon   F3   200.00      444.25     635.50   860.00     1102.25  2260.00
```

```
# Disturbed Trail (D)
traildata <-   LightData %>%
  filter(Habitat == "Trail")
aggregate(Lux ~ TimeBlock * Code, data = traildata, summary)
```

```
##   TimeBlock Code Lux.Min. Lux.1st Qu. Lux.Median Lux.Mean Lux.3rd Qu. Lux.Max.
## 1   Morning    D   3260.0      7552.5    15280.0  20039.0     20625.0  56400.0
## 2 Afternoon    D   1380.0      5460.0    10100.0  17762.0     15545.0  83200.0
```

```
# Research Station (I)
stationdata <-   LightData %>%
  filter(Habitat == "Iyarina")
aggregate(Lux ~ TimeBlock * Code, data = stationdata, summary)
```

```
##   TimeBlock Code Lux.Min. Lux.1st Qu. Lux.Median Lux.Mean Lux.3rd Qu. Lux.Max.
## 1   Morning    I     4.00        7.00      15.50    19.50       28.00    43.00
## 2 Afternoon    I     5.00        7.25      20.00    20.50       33.25    37.00
```

### Plotting the data

```
# Plot the data for each site separately using ggplot2
# Boxplots (medians & IQR)
# with reference lines for the high & low light conditions from the arena
# reference lines show means +/- SD

# Forest plot (F)
forest_plot <-
  ggplot(data = forestdata,
         aes(x = Code, y = Lux, fill = TimeBlock)) +
  labs(y = "Illuminance (lx)",
       x = NULL) +

  scale_y_continuous(limits = c(0, 2500), breaks = seq(0, 2500, 500)) +
  scale_x_discrete(labels = c('F1', 'F2', 'F3')) +

  geom_rect(aes(xmin = -Inf, xmax = Inf,
                ymin = treatment_means$lwr[1], ymax = treatment_means$upr[1]),
            color = "lightgrey", alpha = 0.05) +
  geom_rect(aes(xmin = -Inf, xmax = Inf,
                ymin = treatment_means$lwr[2], ymax = treatment_means$upr[2]),
```

```r
            color = "lightgrey", alpha = 0.05) +

  geom_hline(aes(yintercept = treatment_means$mean_lux[1]),
             linetype = "dashed", linewidth = 0.5, alpha = 0.5) +

  geom_hline(aes(yintercept = treatment_means$mean_lux[2]),
             linetype = "twodash", linewidth = 0.5, alpha = 0.5) +

  geom_boxplot() +

  scale_fill_manual(values=c("grey80", "grey40"),
                    name = "Time of Day") +

theme_bw() +
  theme(axis.line = element_line(colour = "black"),
        panel.grid.major = element_blank(),
        panel.grid.minor = element_blank(),
        panel.background = element_blank()) +
  theme(panel.grid = element_blank()) +
  theme(panel.grid = element_blank()) +
  theme(plot.title = element_blank()) +

  theme(axis.title.y = element_text(size = 9, face = "bold")) +
  theme(axis.title.x = element_text(size = 9, face = "bold")) +
  theme(axis.text.x = element_text(size = 8, face = "bold")) +
  theme(axis.text.y = element_text(size = 8, face = "bold")) +
  theme(axis.ticks.y = element_line(linewidth = 0.7)) +
  theme(axis.ticks.x = element_line(linewidth = 0.7)) +

  theme(legend.position = c(0.11, 0.83)) +
  theme(legend.title = element_text(size = 6, face = "bold")) +
  theme(legend.text = element_text(size = 6, face = "bold")) +
  theme(legend.key = element_rect(color = NA, fill = NA),
        legend.key.size = unit(0.3, "cm"))
```

```
## Warning: A numeric `legend.position` argument in `theme()` was deprecated in ggplot2
## 3.5.0.
## i Please use the `legend.position.inside` argument of `theme()` instead.
## This warning is displayed once every 8 hours.
## Call `lifecycle::last_lifecycle_warnings()` to see where this warning was
## generated.
```

```r
###
# Disturbed Trail plot (D)
trail_plot <-
  ggplot(data = traildata,
         aes(x = Code, y = Lux, fill = TimeBlock)) +
  labs(y = "Illuminance (lx)",
       x = NULL) +

  scale_y_continuous(limits = c(0, 84000), breaks = seq(0, 80000, 20000)) +
  scale_x_discrete(labels = c('D')) +

  geom_rect(aes(xmin = -Inf, xmax = Inf,
```

```r
                ymin = treatment_means$lwr[1], ymax = treatment_means$upr[1]),
            color = "lightgrey", alpha = 0.05) +
  geom_hline(aes(yintercept = treatment_means$mean_lux[1]),
             linetype = "dashed", linewidth = 0.5, alpha = 0.5) +

  geom_rect(aes(xmin = -Inf, xmax = Inf,
                ymin = treatment_means$lwr[2], ymax = treatment_means$upr[2]),
            color = "lightgrey", alpha = 0.05) +
  geom_hline(aes(yintercept = treatment_means$mean_lux[2]),
             linetype = "twodash", linewidth = 0.5, alpha = 0.5) +

  geom_boxplot() +

  scale_fill_manual(values=c("grey80", "grey40"),
                    name = "Time of Day") +

  theme_bw() +
  theme(axis.line = element_line(colour = "black"),
        panel.grid.major = element_blank(),
        panel.grid.minor = element_blank(),
        panel.background = element_blank()) +
  theme(panel.grid = element_blank()) +
  theme(panel.grid = element_blank()) +
  theme(plot.title = element_blank()) +

  theme(axis.title.y = element_text(size = 9, face = "bold")) +
  theme(axis.title.x = element_text(size = 9, face = "bold")) +
  theme(axis.text.x = element_text(size = 8, face = "bold")) +
  theme(axis.text.y = element_text(size = 8, face = "bold")) +
  theme(axis.ticks.y = element_line(linewidth = 0.7)) +
  theme(axis.ticks.x = element_line(linewidth = 0.7)) +

  theme(legend.position = c(0.25, 0.83)) +
  theme(legend.title = element_text(size = 6, face = "bold")) +
  theme(legend.text = element_text(size = 6, face = "bold")) +
  theme(legend.key = element_rect(color = NA, fill = NA),
        legend.key.size = unit(0.3, "cm"))

###
# Research Station Plot (I)
station_plot <-
  ggplot(data = stationdata,
         aes(x = Code, y = Lux, fill = TimeBlock)) +
  labs(y = "Illuminance (lx)",
       x = NULL) +

  scale_y_continuous(limits = c(0, 150), breaks = seq(0, 150, 30)) +
  scale_x_discrete(labels = c('I')) +

  geom_rect(aes(xmin = -Inf, xmax = Inf,
                ymin = treatment_means$lwr[2], ymax = treatment_means$upr[2]),
            color = "lightgrey", alpha = 0.05) +
```

```r
  geom_hline(aes(yintercept = treatment_means$mean_lux[2]),
             linetype = "twodash", linewidth = 0.5, alpha = 0.5) +

  geom_boxplot() +

  scale_fill_manual(values=c("grey80", "grey40"),
                    name = "Time of Day") +

  theme_bw() +
  theme(axis.line = element_line(colour = "black"),
        panel.grid.major = element_blank(),
        panel.grid.minor = element_blank(),
        panel.background = element_blank()) +
  theme(panel.grid = element_blank()) +
  theme(panel.grid = element_blank()) +
  theme(plot.title = element_blank()) +

  theme(axis.title.y = element_text(size = 9, face = "bold")) +
  theme(axis.title.x = element_text(size = 9, face = "bold")) +
  theme(axis.text.x = element_text(size = 8, face = "bold")) +
  theme(axis.text.y = element_text(size = 8, face = "bold")) +
  theme(axis.ticks.y = element_line(linewidth = 0.7)) +
  theme(axis.ticks.x = element_line(linewidth = 0.7)) +

  theme(legend.position = c(0.25, 0.83)) +
  theme(legend.title = element_text(size = 6, face = "bold")) +
  theme(legend.text = element_text(size = 6, face = "bold")) +
  theme(legend.key = element_rect(color = NA, fill = NA),
        legend.key.size = unit(0.3, "cm"))

# Combining the plots together into one multifaceted figure
# Specify the layout
layout <- "AA
          BC"
# Combine the plots
combined_plot_lighting <- forest_plot + trail_plot + station_plot +
  plot_layout(design = layout)
print(combined_plot_lighting)
```

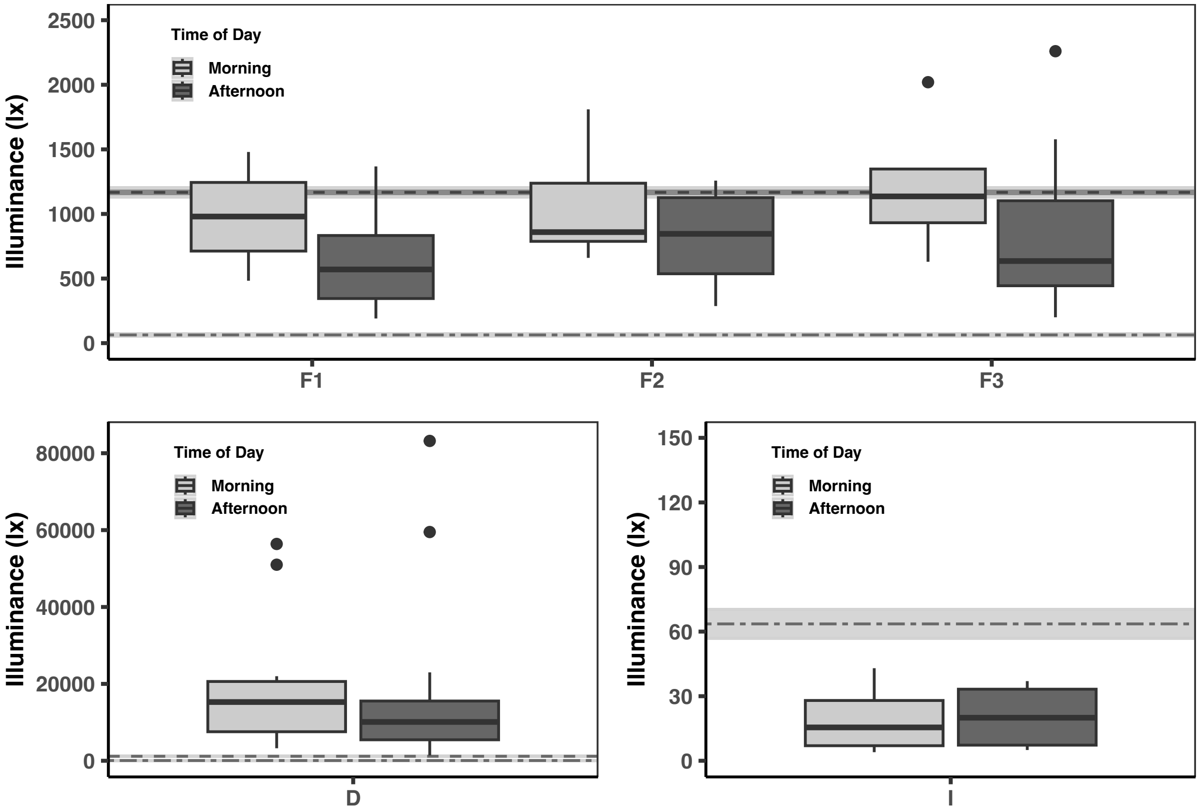


```
# Save plots as Figure S1
jpeg(filename="FigS1_LightEnvironment.jpeg",
     width = 120, height=120, units='mm', bg='white', res=300)
plot(combined_plot_lighting)
dev.off()
```

```
## pdf
##   2
```
