# Supplementary material for: Larger frogs are better mimics but are more risk-averse in a nontoxic poison frog
Source: Behav Ecol. 2025 Oct 6;36(6):araf117. doi: 10.1093/beheco/araf117 (PMC12596420; doi:10.1093/beheco/araf117)
Supplement: araf117_Supplementary_Data [file araf117_supplementary_data.zip › McEwen-etal-2025_SupplementaryMaterial.pdf]

Supplementary Material for:

LARGER FROGS ARE BETTER MIMICS BUT ARE MORE RISK-AVERSE IN A NON-TOXIC POISON FROG

Brendan L. McEwen, Justin Yeager, Ana Veneat, & James B. Barnett

Quantifying the light environment

Methods

To contextualize the two light intensity treatments used in the behavioural assays, in August (12th – 21st) 2024, we surveyed light intensity (lux) within the experimental arena and in the frogs' natural habitat at the Iyarina Forest Reserve. All measurements were taken with a Digital LED Light Meter (Dr.meter LX1332B, USA) and performed by the same researcher (BLM).

To quantify the two lighting treatments (*high* and *low*), we recorded the illuminance found at the centre of the experimental arena with the light meter held 30 cm from the light source. These measurements were taken on a single day, at ~1-hour increments between 09:30 am and 15:30 pm (n = 7 per treatment). We then calculated the mean lux ± standard deviation for the two conditions.

To quantify natural levels of illumination, we conducted light intensity surveys at three locations, close to where *Al. zaparo* had been encountered, within the Iyarina Forest Reserve: 1) three sites within the enclosed canopy forest (F1-3), 2) one site along a human-constructed trail into the forest (D), and 3) one site located among the research station buildings (I). At each site, we measured light intensity by holding the light meter at waist height, slowly rotating 360°, and recording the highest intensity registered during the arc. Measurements were taken under a variety of natural lighting conditions, but weather conditions were consistent across all sites within each sampling day.

We recorded light intensity at three sites within the closed-canopy forest (F1-3), where *Al. zaparo* had been observed calling. Each of the three sites was measured at five sub-sites: at the point where the frog was first spotted, and 10 m to the north, south, east, and west of that central location. Sites F1 and F2 were measured four times (two mornings and two afternoons) whereas site F3 was measured three times (one morning and two afternoons). We also recorded light intensity along a human-cleared trail leading into the forest (D). Here, we measured five sub-sites spaced 10 m apart along the first 50 m of the trail leading from the access road and into the forest (Da-e, 10 m – 50 m along the trail). The trail was surveyed five times (two mornings and three afternoons). Morning and afternoon surveys were conducted between 09:00 am - 12:30 pm and 14:00 pm – 16:30 pm, respectively.

To measure light intensity at the beginning and the end of the frog's daily activity cycle (i.e., when light levels were expected to be lowest) we listened for the first and last bouts of male calling at one site around the field station buildings (I). Acoustic surveys were conducted on two separate mornings and two separate afternoons with light measurements being taken from the location of the first (morning) and last (afternoon) calling male. Two measurements were taken from the site of each frog: 1) under an open/broken canopy and 2) from beneath a closed canopy. Morning surveys began at 06:00 am and continued until the first *Al. zaparo* was heard calling (~06:30 am). Afternoon surveys began at 18:00 pm and continued until no *Al. zaparo* call had been heard for 5 minutes (~18:30 pm).

Results

Our data suggest that the experimental lighting treatments used in the behavioural arena (*high* and *low*) reasonably approximated an ecologically plausible range of illumination, that was close to both the highest and lowest light intensities found at known *Al. zaparo* calling sites (Fig. S1).

We found that light intensity within the behavioural arena during the *high* light condition (mean ± SD: *high* = 1167.29 ± 33.73 lx) was similar to the range of light intensities recorded within the enclosed canopy forest sites (median (IQR): F1: morning = 980 lx (713-1244), afternoon = 571 lx (345-833); F2: morning = 860 lx (788-1238), afternoon = 846 lx (537-1126); F3: morning = 1136 lx (932-1348), afternoon = 636 lx (444-1102); Fig S1 top). This value was considerably lower than the illumination recorded along the broken-canopy trail (median (IQR): D: morning = 15280 lx (7553-20625), afternoon = 10100 lx (5460-15545); Fig S1 bottom left). Whereas the light intensity of the low light treatment (mean ± SD: *Low* = 63.57 ± 6.75 lx) was similar to that recorded at the research station site (I) during the morning onset and evening conclusion of frog calling behaviour (median (IQR): I: morning = 16 lx (7-28), afternoon = 20 lx (7-33); Fig S1 bottom right).

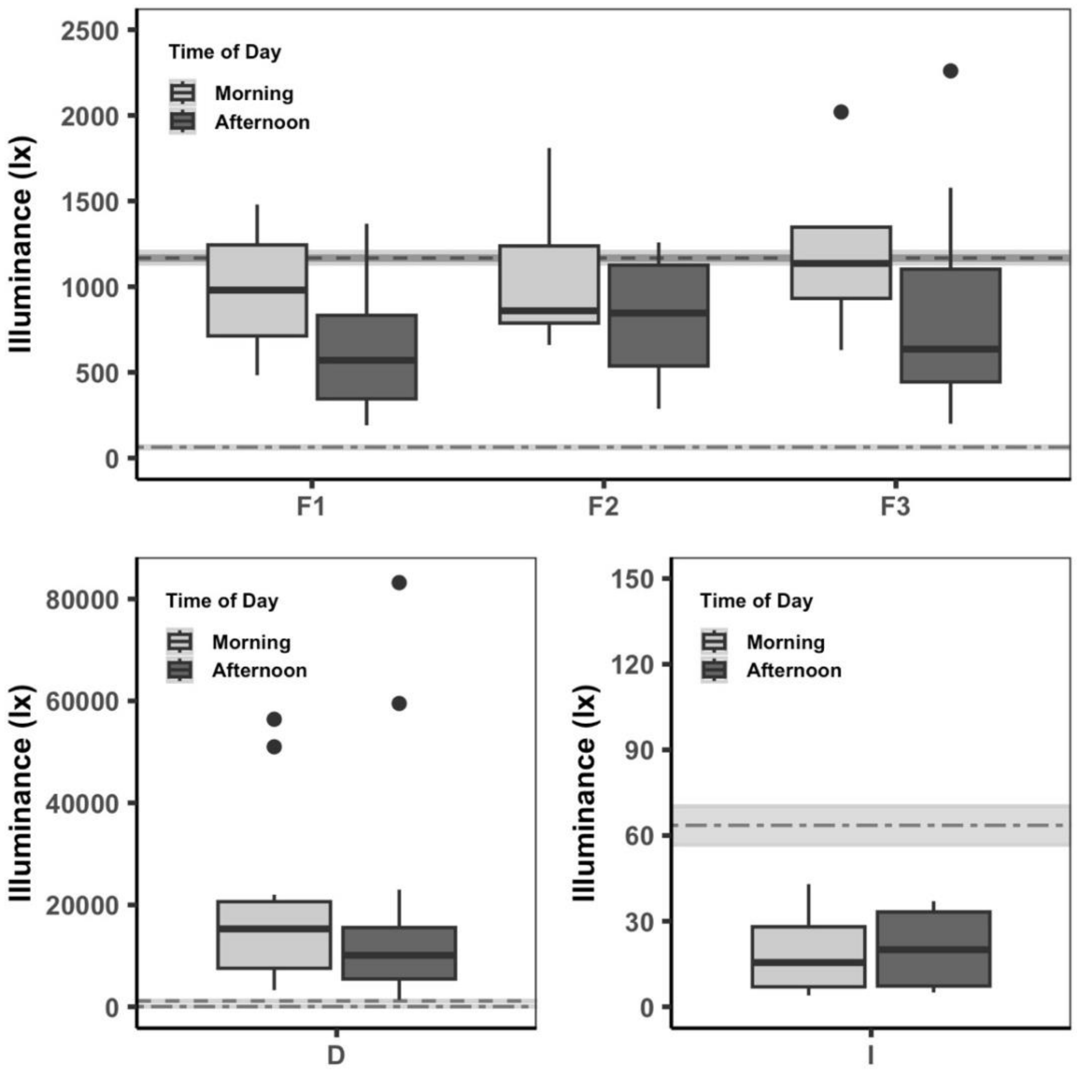

Figure S1. Illumination (lx) for each location (boxplots: F = forest; D = disturbed trail; I = Iyarina research station) during the morning (light grey) and afternoon (dark grey) relative to the *low* (lower dot-dashed line, mean ± SD) and *high* (upper dashed line, mean ± SD) light conditions used in the experimental arena. Experimental conditions are within the range of light conditions experienced by frogs in their natural habitat. Top: illumination in the closed canopy forest is comparable to the *high* light condition. Bottom left: illumination along the disturbed, broken canopy, trail is much higher than both experimental conditions. Bottom right: illumination at the beginning (morning, light grey) and conclusion (afternoon, dark grey) of frog activity is comparable to the *low* light condition (only the *low* light treatment (dot-dashed line) is plotted at this scale).
